# From diffusive mass transfer in Stokes flow to low Reynolds number Marangoni boats

**DOI:** 10.1140/epje/s10189-021-00034-9

**Published:** 2021-02-12

**Authors:** Hendrik Ender, Jan Kierfeld

**Affiliations:** Department of Physics, Technische Universität Dortmund, 44221 Dortmund, Germany

## Abstract

**Abstract:**

We present a theory for the self-propulsion of symmetric, half-spherical Marangoni boats (soap or camphor boats) at low Reynolds numbers. Propulsion is generated by release (diffusive emission or dissolution) of water-soluble surfactant molecules, which modulate the air–water interfacial tension. Propulsion either requires asymmetric release or spontaneous symmetry breaking by coupling to advection for a perfectly symmetrical swimmer. We study the diffusion–advection problem for a sphere in Stokes flow analytically and numerically both for constant concentration and constant flux boundary conditions. We derive novel results for concentration profiles under constant flux boundary conditions and for the Nusselt number (the dimensionless ratio of total emitted flux and diffusive flux). Based on these results, we analyze the Marangoni boat for small Marangoni propulsion (low Peclet number) and show that two swimming regimes exist, a diffusive regime at low velocities and an advection-dominated regime at high swimmer velocities. We describe both the limit of large Marangoni propulsion (high Peclet number) and the effects from evaporation by approximative analytical theories. The swimming velocity is determined by force balance, and we obtain a general expression for the Marangoni forces, which comprises both direct Marangoni forces from the surface tension gradient along the air–water–swimmer contact line and Marangoni flow forces. We unravel whether the Marangoni flow contribution is exerting a forward or backward force during propulsion. Our main result is the relation between Peclet number and swimming velocity. Spontaneous symmetry breaking and, thus, swimming occur for a perfectly symmetrical swimmer above a critical Peclet number, which becomes small for large system sizes. We find a supercritical swimming bifurcation for a symmetric swimmer and an avoided bifurcation in the presence of an asymmetry.

**Graphic abstract:**

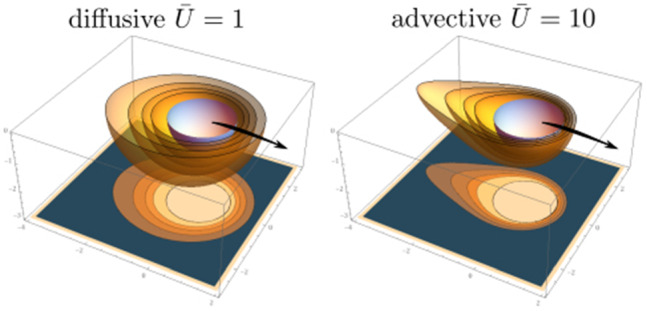

## Introduction

Swimming on the microscale is governed by low Reynolds numbers and requires special propulsion mechanisms which are effective in the presence of dominating viscous forces. An important class of low Reynolds number swimming strategies generates interfacial fluid slip-velocities at the swimmer surface, which then lead to self-propulsion because the swimmer must be force-free. This class of swimming strategies comprises phoretic and Marangoni mechanisms. Phoretic mechanisms self-create gradients in concentration (self-diffusiophoresis) or temperature (self-thermophoresis) [1, 2] which, in turn, give rise to interfacial fluid flow in a thin interaction layer [3].

There are two types of swimmers based on the Marangoni effect [4]: droplet swimmers with liquid interfaces, which can operate in the bulk and solid Marangoni boats or surfers operating at a liquid–air interface. The liquid droplet swimmer is fully immersed in a liquid that carries surfactant. Propulsion is generated by the Marangoni effect, which creates a slip velocity from a surfactant concentration gradient along the entire liquid–liquid interface between swimmer and surrounding liquid. One typical mechanism to maintain such a surfactant gradient is that more surfactant is adsorbed at the front (in swimming direction) of the swimmer, which depresses the interfacial tension in the front [5–7]. In Ref. [8], an auto-diffusiophoretic mechanism coupled to advection [9, 10] has been proposed to maintain the surfactant concentration gradient. This propulsion mechanism based on the Marangoni effect is utilized in different liquid Marangoni swimmers, for example, active liquid droplets or active emulsions [6], such as pure water droplets in an oil–surfactant medium (squalane and monoolein) [8] or liquid crystal droplets in surfactant solutions [6]. Many liquid Marangoni swimmers are spherically symmetric initially and swimming spontaneously breaks this symmetry. Beyond the instability, advection and/or preferred adsorption can produce sufficiently strong surfactant concentration gradients and swimming velocities to maintain advection and/or preferred adsorption [5, 7, 9]. Also, asymmetric shape changes can give rise to concentration gradients and sufficient swimming velocities to maintain asymmetric shapes [11, 12].

Here, we consider Marangoni boats or surfers, which employ a different propulsion mechanism. Important examples are soap or camphor boats which have a long history [13]. The crucial difference to liquid Marangoni swimmers is that these boats or surfers operate at a liquid–air interface rather than in the bulk of the liquid. Propulsion is not caused by surfactants that are anisotropically distributed along the swimmer–liquid interface but by the anisotropic distribution of surfactant at the liquid–air interface along which the soap boat propels [14]. The surfactant molecules at the liquid–air interface are emitted or dissolved from the swimmer; this can be achieved by depositing them on the floating swimmer initially [15], by soaking the swimmer in surfactant [16–21], or by using a swimmer body made from dissolving surfactant [22]. There are many examples based on DMF (dimethylformamide) [23], alcohol [15, 21], soap [21], camphor [16–20, 24] or camphene [22] that have also been investigated quantitatively. In a companion paper [25], we discuss alginate capsules as versatile interfacial Marangoni swimmers working with many surface tensions reducing “fuels” in detail, in particular, polyethylene glycol (PEG)-loaded alginate capsules.

So far, Marangoni boats can be produced down to radii $a\sim 150\,{\upmu \mathrm{m}}$ [25], and quantitative results are available down to $a\sim 1500\,{\upmu \mathrm{m}}$ with Reynolds numbers $\mathrm{Re} \sim 60$, which is still above the low Reynolds number regime. Miniaturization is approaching the low Reynolds number regime, which is the regime we address in detail in the present paper. In a companion paper [25], we discussed low Reynolds number results more briefly and aimed to generalize to high Reynolds numbers using the concept of the Nusselt number in order to describe experiments on PEG–alginate capsule swimmers and camphor boats quantitatively. There is a related system of thermal Marangoni surfers propelled by the thermal Marangoni effect, which was successfully realized only recently [26]. Its theoretical description is equivalent to surfactant-driven Marangoni boats with thermal advection–diffusion replacing surfactant advection-diffusion. Because thermal diffusion coefficients are much higher and swimmer radii reach down to micrometers, this system operates at low Reynolds numbers.

The surfactant molecules are emitted or dissolved from the Marangoni boat, diffuse and advect to fluid flow in the water phase and adsorb to the air–water interface, eventually in interplay with evaporation for volatile surfactants. This creates surface tension gradients and Marangoni stresses on the fluid. Surface tension gradients give rise to a direct net propulsion force (*direct Marangoni force* in the following). Marangoni stresses on the fluid give rise to symmetry-broken Marangoni flows, which also contribute to (or impede) propulsion via hydrodynamic drag onto the swimmer (*Marangoni flow forces* in the following). Direct Marangoni forces propel into the direction of *higher* surface tension, i.e., *lower* surfactant concentration along the air–water–swimmer contact line. We note that this is *opposite* to the propulsion in the direction of higher surfactant concentration for the liquid Marangoni swimmers operating in the bulk [5–9].

**Fig. 1:**
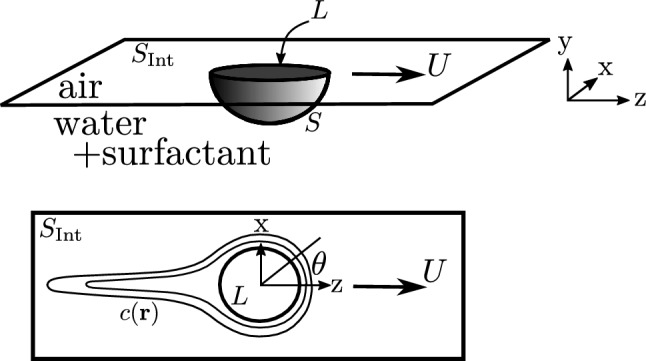
Side view (top) and top view (bottom) of the half-spherical Marangoni swimmer geometry with surfactant concentration field $c(\varvec{r})$ and coordinates

A full quantitative theory of Marangoni boats including hydrodynamics, surfactant advection, direct Marangoni forces and Marangoni flows is still elusive but numerical approaches exist [14, 18, 27, 28]. Theoretical approaches ignore the advection of the surfactant concentration field [29–31] or even ignore hydrodynamic flow fields [16, 17, 24, 32] or approximate it by uniform flow [20], which clearly oversimplifies the description of surfactant transport. Here, we focus on low Reynolds numbers as in Refs. [27, 29–31] and consider a half-spherical swimmer geometry (see Fig. 1), which can simplify the theoretical treatment because axial symmetry can be exploited in certain limits. Experimentally, half-spherical swimmers can be fabricated using the PEG–alginate system [25]. In the limit of weak Marangoni flows, for example, the fluid flow reduces to the well-known Stokes flow around a sphere. We fully include advection of the surfactant concentration field into our analysis as opposed to Refs. [29–31], where disks and spheres propelled by the soap boat mechanism have been considered previously. If advection is ignored, the formation of a concentration boundary layer at higher velocities, as it is well known from the related problem of mass transfer from a sphere in laminar Stokes flow [33–36], will be missed. This happens for velocities $U\gg a/D$, where *a* is the sphere radius and *D* the surfactant diffusion constant, and is essential for the resulting swimming velocity, which is the quantity of main interest in this paper.

In order to obtain the Marangoni forces onto the swimmer, we have to calculate the surfactant concentration profile and have to revisit the problem of mass transfer from a sphere in laminar Stokes flow both for constant concentration and constant flux boundary conditions. In particular, analytical results on the relevant flux boundary conditions are missing in the literature. We fill this gap and derive results for concentration profiles and for the angular dependence of the Nusselt number both for isotropic and anisotropic emission. This allows us to calculate Marangoni propulsion forces both in the diffusive and in the advection-dominated regime and provide analytical results. There is a direct Marangoni force, which is propelling the swimmer into the direction of higher surface tension and a Marangoni flow force, as has been worked out by Masoud and Stone [37]. We will unravel, under which conditions the flow force contribution is propelling or dragging the swimmer. We can extend our analysis to large Marangoni propulsion (high Peclet number) and include effects from evaporation by approximative analytical theories. This part of the analysis is also the focus of a companion paper [25]. Therefore, this discussion is shortened in this paper. All analytical results are corroborated by numerical finite element calculations employing a novel iterative approach.

As opposed to previous work [14, 18, 27–31], we consider here completely symmetric Marangoni boats with isotropic surfactant emission as motivated by the experiments in Ref. [25]. Swimming is established in a symmetry-breaking bifurcation. Similar swimming bifurcations have been analyzed by Michelin and coworkers [5, 7–10], but for a different type of swimmer, namely liquid droplet bulk Marangoni swimmers. For symmetric surface swimmers propelled by thermal Marangoni forces, a related symmetry-breaking effect has been observed in Ref. [38], where symmetric microbeads spontaneously circle around a heating laser beam. Our analysis allows us to obtain the swimming velocity of Marangoni boats as a function of Marangoni propulsion strength (Peclet number) and to analyze in detail the nature of the symmetry-breaking swimming bifurcation.

## Model

We introduce coordinates such that the origin $r=0$ is at the center of the planar surface of the half-sphere, the liquid–air interface is at $y=0$ (with $y<0$ being the liquid phase), and $\varvec{e}_z$ will coincide with the spontaneously selected swimming direction, see Fig. 1. We also use spherical coordinates such that $\theta = 0$ is the swimming direction and the interfacial plane is located at $\phi = 0,\pi$ ($y=0$). The half-sphere has radius *a* such that the contact line is at $r=a$ and $\phi = 0,\pi$ (and parameterized by $\theta$). We denote the half-spherical surface of the swimmer by *S*, the circular air–water–swimmer contact line by *L*, and the liquid–air interface outside the swimmer as $S_{\mathrm{Int}}$.

The general strategy to determine the swimming speed has been outlined in Ref. [25]. We first prescribe a stationary velocity $\varvec{U} = U \varvec{e}_z$ of the swimmer and analyze the following three coupled problems for its stationary state:

(i) Surface tension reduction by surfactant adsorption at the air–water interface; depending on the volatility of the surfactant, we also need to include evaporation.
(ii) Low Reynolds number fluid flow including both Stokes flow around the half-sphere and additional surfactant-induced fluid Marangoni flow.
(iii) Diffusive surfactant release or surfactant dissolution from the swimmer and subsequent diffusion and advection.

We will start from the diffusion–advection problem (iii) in the presence of Stokes flow around a sphere and neglecting Marangoni flow. This will also give new results for the mass transfer from spheres in laminar Stokes flow. We later examine the additional effects of Marangoni flow and evaporation. The fully coupled problems can also be treated numerically.

Solving these three coupled problems, we can obtain the Marangoni forces as a function of the prescribed velocity *U* from the surfactant concentration profile by employing the reciprocal theorem. Finally, the actual swimming velocity $U=U_{\mathrm{swim}}$ is determined from the condition of a force-free swimmer, i.e., the force equilibrium between Stokes drag force, direct propelling Marangoni forces from the surface tension gradient along the air–water–swimmer contact line and Marangoni flow forces.

We begin with a short recapitulation of the governing equations [25].

### Coupled adsorption, fluid flow and diffusion–advection problems

Regarding sub-problem (i), we use a local and linear relationship for the surface tension reduction

1 $$\begin{aligned} \varDelta \gamma (\varvec{r})&= -\kappa c(\varvec{r}) \end{aligned}$$

($\varvec{r}$ is an interfacial vector with $y=0$) by the local surfactant concentration difference $c(\varvec{r})$ with respect to a bulk concentration background value $c_0$ (the concentration at $|\varvec{r}|\rightarrow \infty$); $\kappa$ is a coefficient characterizing the propulsion strength. In formulating Eq. (1) locally, we assumed fast on and off kinetics of surfactant to the interface [39] such that the interfacial concentration $\varGamma (\varvec{r})$ is slaved to the bulk and only a passive “reporter” of the bulk subsurface concentration $\left. c(\varvec{r})\right| _{y=0}$. This is appropriate for water-soluble surfactants but, for example, the opposite limit of what has been considered in Refs. [29, 31], where surfactant is strictly confined to the interface.

Fast on and off kinetics also implies that an imbalance of flux to and from the interface into the bulk can only arise from an additional evaporating flux from the interface to the gas phase. In the general case including surfactant evaporation from the interface, the balance of fluxes to and from the interface gives

2 $$\begin{aligned} j_{\mathrm{Int}} = -\left. D \varvec{\nabla } c(\varvec{r})\cdot \varvec{n}^{\mathrm{out}}\right| _{y=0} = - j_{\mathrm{ev}} = k\left. c(\varvec{r})\right| _{y=0}, \nonumber \\ \end{aligned}$$

where *k* is the rate constant for evaporation. This provides the boundary condition to the diffusion–advection sub-problem (iii) in the bulk.

Regarding the low Reynolds number fluid flow sub-problem (ii), we consider the rest frame of the swimmer and linearly decompose the total fluid flow field into a field $\varvec{v}(\varvec{r})$, which is the flow field of a half-sphere pulled with velocity $U\varvec{e}_z$ through the liquid and a correction $\varvec{{v}}_{\mathrm{M}}(\varvec{r})$ from Marangoni flows, $\varvec{v}_{\mathrm{tot}}(\varvec{r}) = \varvec{v}(\varvec{r}) + \varvec{{v}}_{\mathrm{M}}(\varvec{r})$. For low Reynolds numbers, both $\varvec{v}(\varvec{r})$ and $\varvec{{v}}_\mathrm{M}(\varvec{r})$ (and the associated pressure fields) fulfill the incompressibility condition $\varvec{\nabla }\cdot \varvec{v} = 0$ and the linear Stokes equation $\mu \varvec{\nabla }^2 \varvec{v} = \varvec{\nabla } p$, where $\mu$ is the fluid viscosity.

The flow field $\varvec{v}(\varvec{r})$ of an externally pulled half-sphere is given by “half” ($y<0$) the Stokes flow field around a sphere, which automatically fulfills the boundary condition $\left. v_y(\varvec{r}) \right| _{y=0} = 0$ for symmetry reasons. In spherical coordinates, the axisymmetric Stokes flow field is

3a $$\begin{aligned} \varvec{v}(\varvec{r})&= {\hat{u}}(r,\theta ) \varvec{e}_r + {\hat{v}}(r,\theta ) \varvec{e}_\theta ~~\text{ with } \nonumber \\ {\hat{u}}(r,\theta )&= U \cos \theta \left[ -\frac{1}{2} \left( \frac{a}{r}\right) ^3 + \frac{3}{2} \frac{a}{r} - 1 \right] \equiv U \cos \theta u(r/a), \end{aligned}$$

3b $$\begin{aligned} {\hat{v}}(r,\theta )&= U \sin \theta \left[ -\frac{1}{4} \left( \frac{a}{r}\right) ^3 - \frac{3}{4} \frac{a}{r} + 1 \right] \equiv U \sin \theta v(r/a). \end{aligned}$$

The total flow field $\varvec{v}_{\mathrm{tot}}(\varvec{r})$ also has no-slip boundary conditions on the surface of the sphere and assumes $\varvec{v}_{\mathrm{tot}}(\infty ) = - U\varvec{e}_z$ at infinity, but is subject to Marangoni stresses at the liquid–air interface. Consequently, the difference $\varvec{v}_{\mathrm{M}}(\varvec{r}) = \varvec{v}_{\mathrm{tot}}(\varvec{r})- \varvec{v}(\varvec{r})$ from Marangoni flows has no-slip boundary conditions on the surface of the sphere, has vanishing velocity $\varvec{v}_{\mathrm{M}}(\infty ) = 0$ at infinity and is subject to Marangoni stresses at the liquid–air interface. Moreover, for all three flow fields, there is no normal flow across the liquid–air interface. We will assume that the liquid–air interface remains flat, even if the sphere moves. This requires that typical viscous forces remain small compared to interfacial stress, $\mu U \ll \gamma$, which is fulfilled with $\mu U\,\sim \,10^{-5}\,\mathrm{N/m}$ for generic Marangoni boats with $U\sim 1\,\mathrm{cm/s}$ and $\gamma \sim 0.07\,\mathrm{N/m}$ for the air–water interface. We also neglect a possible curvature of the interface from wetting effects.

The Marangoni flow is caused by tangential Marangoni stresses at the liquid–air interface $y=0$,

4 $$\begin{aligned} \mu \varvec{n}^{\mathrm{out}}\cdot \varvec{\nabla } \left. \varvec{v}_{\mathrm{M}}(\varvec{r})\right| _{y=0} = \mu \partial _y \left. \varvec{v}_{\mathrm{M}}(\varvec{r})\right| _{y=0} = \varvec{\nabla }_S \varDelta \gamma (\varvec{r}),\nonumber \\ \end{aligned}$$

which act both on $\varvec{v}_{\mathrm{M}}$ and $\varvec{v}_{\mathrm{tot}}$.

Surfactant diffusion and advection (iii) will play a central role. Surfactant molecules are emitted from the half-spherical surface *S* and diffuse in the liquid phase. At the same time, they are advected by the total fluid flow. In the stationary state, the bulk concentration field is governed by the diffusion–advection equation

5 $$\begin{aligned} 0= \partial _t c&= D \varvec{\nabla }^2 c - (\varvec{v}(\varvec{r})+ \varvec{v}_{\mathrm{M}}(\varvec{r})) \cdot \varvec{\nabla } c. \end{aligned}$$

We consider two types of boundary conditions which seem most important for applications: (A) slow diffusional surfactant release on *S* leading to a constant flux boundary condition or (B) surfactant dissolution from the swimmer or surfactant production by some chemical reaction by the swimmer leading to a constant concentration boundary condition,

6 $$\begin{aligned} \text{(A) } \text{ constant } \text{ flux: }~~&\left. \varvec{j}\cdot \varvec{n}\right| _S&=- D\left. \varvec{\nabla } c\cdot \varvec{n} \right| _S =\alpha , \end{aligned}$$

7 $$\begin{aligned} \text{(B) } \text{ constant } \text{ conc.: }~~&\left. c \right| _S&= c_S \end{aligned}$$

together with $c(\infty )=0$ and the no-flux boundary condition at the interface $S_{\mathrm{Int}}$. The surface flux $\alpha$ or the surface concentration $c_S$ is assumed to be only slowly changing on the time scales of the fluid flow and the surfactant diffusion and approximated as a constant for the calculation of quasi-stationary fluid flow and concentration fields.

We non-dimensionalize sub-problems (i)–(iii) by measuring lengths in units of *a*, velocities in units of *D*/*a*, concentrations in units of $\alpha a/D$

8 $$\begin{aligned} \varvec{\rho }&\equiv \varvec{r}/a,~~\bar{\varvec{\nabla }} \equiv a \varvec{\nabla } = \varvec{\nabla }_\rho , ~~\bar{\varvec{v}}\equiv \varvec{v} \frac{a}{D},~~ {\bar{U}} \equiv U \frac{a}{D}, \nonumber \\ {\bar{c}}&\equiv c \frac{D}{\alpha a},~~ {\bar{j}} \equiv j\frac{1}{\alpha }, ~~{\bar{p}} \equiv p \frac{a^2}{D\mu }. \end{aligned}$$

The prescribed dimensionless velocity ${\bar{U}}$ of the swimmer is the first control parameter of the problem,^1^ which is related to the Reynolds number, $\mathrm{Re} = 2{\bar{U}}/\mathrm{Sc}$, via the Schmidt number $\mathrm{Sc} \equiv \mu /\rho D$. Low Reynolds numbers $\mathrm{Re}\ll 1$ are realized for ${\bar{U}} \ll \mathrm{Sc}/2$, which can still be much larger than unity as typical Schmidt numbers for surfactants in aqueous solutions are of the order of 1000. Therefore, we have to discuss *both* the diffusive case ${\bar{U}}\ll 1$ *and* the advective case ${\bar{U}}\gg 1$, even at low Reynolds numbers.

**Table 1:** Dimensionless parameters. $\mathrm{Re}$ or ${\bar{U}}$, $\mathrm{Sc}$, $\mathrm{Pe}$, and ${\bar{k}}$ are control parameters of the problem

Dimensionless parameter	Formula	Eqs.
Reynolds number $\mathrm{Re}$	$={\rho U 2a}/{\mu } = {2{\bar{U}}}/{\mathrm{Sc}}$	
dimensionless velocity ${\bar{U}}$	$= U{a}/{D}$	
Schmidt number $\mathrm{Sc}$	$= {\mu }/{\rho D}$	
Peclet number $\mathrm{Pe}$	$= {\kappa \alpha a^2}/{D^2 \mu }$	(10)
Biot number ${\bar{k}}$	$= {ak}/{D}$	(11)
swimming velocity ${\bar{U}}_{\mathrm{swim}}$	$= U_{\mathrm{swim}}{a}/{D}$	(16)
Marangoni Reynolds number $\mathrm{Re}_{\mathrm{M}}$	$= {2\mathrm{Pe}}/{\mathrm{Sc}}$	
Nusselt (or Sherwood) number $\mathrm{Nu}$ ($\mathrm{Sh}$)	$={-\partial _\rho {\bar{c}}_0(1)}/{{\bar{c}}_0(1)}$	(20)

$\mathrm{Re}_{\mathrm{M}}$ and $\mathrm{Nu}$ cannot be independently controlled but characterize the resulting solutions; the swimming velocity ${\bar{U}}_{\mathrm{swim}}$ is determined by the force balance swimming condition

Our dimensionless set of equations for problems (i)–(iii) becomes

9a $$\begin{aligned}&\mathrm{(i)}\qquad -\left. \bar{\varvec{\nabla }} {\bar{c}}(\varvec{\rho })\cdot \varvec{n}^{\mathrm{out}} \right| _{{\bar{y}}=0}\approx 0\nonumber \\&\quad \text{ without } \text{ evaporation, } \end{aligned}$$

9b $$\begin{aligned}&\quad -\left. \bar{\varvec{\nabla }} {\bar{c}}(\varvec{\rho })\cdot \varvec{n}^{\mathrm{out}} \right| _{{\bar{y}}=0} \approx {\bar{k}}\left. {\bar{c}}(\varvec{\rho }) \right| _{{\bar{y}}=0} \nonumber \\&\quad \text{ with } \text{ evaporation, } \end{aligned}$$

9c $$\begin{aligned}&\mathrm{(ii)}\qquad \bar{\varvec{v}}_{\mathrm{tot}}(\varvec{\rho }) = \bar{\varvec{v}}(\varvec{\rho }) + \bar{\varvec{v}}_{\mathrm{M}}(\varvec{\rho }), \nonumber \\&\mathrm{(iia)}\qquad \bar{\varvec{v}}(\rho ,\theta ) = {\bar{U}}\cos \theta u(\rho ) \varvec{e}_r + {\bar{U}} \sin \theta v(\rho ) \varvec{e}_\theta \nonumber \\&\quad \text{ Stokes } \text{ flow } \text{ field, } \end{aligned}$$

9d $$\begin{aligned}&\mathrm{(iib)}\qquad \bar{\varvec{\nabla }}\cdot \bar{\varvec{v}}_{\mathrm{M}} = 0\nonumber \\&\quad \text{ Marangoni } \text{ flow } \text{ field, } \nonumber \\&\bar{\varvec{\nabla }}^2 \bar{\varvec{v}}_{\mathrm{M}} = \bar{\varvec{\nabla }} {\bar{p}}_{\mathrm{M}}, \nonumber \\&\bar{\varvec{v}}_{\mathrm{M}}(\infty ) =0, \nonumber \\&\quad \left. \bar{\varvec{v}}_{\mathrm{M}}(\varvec{\rho }) \right| _{\rho =1}=0, \nonumber \\&\quad \left. {\bar{v}}_{\mathrm{M, y}}(\varvec{\rho }) \right| _{{\bar{y}}=0} =0, \nonumber \\&\quad \left. \partial _{{\bar{y}}} \bar{\varvec{v}}_{\mathrm{M}}(\varvec{\rho }) \right| _{{\bar{y}}=0} = -\mathrm{Pe} \left. \bar{\varvec{\nabla }}_{S} {\bar{c}}(\varvec{\rho }) \right| _{{\bar{y}}=0}, \end{aligned}$$

9e $$\begin{aligned}&\mathrm{(iii)}\qquad 0 = \bar{\varvec{\nabla }}^2 {\bar{c}} - (\bar{\varvec{v}}(\varvec{\rho })+ \bar{\varvec{v}}_{\mathrm{M}}(\varvec{\rho })) \cdot \bar{\varvec{\nabla }} {\bar{c}}, \end{aligned}$$

9f $$\begin{aligned}&{\bar{c}}(\infty ) = 0, \nonumber \\&\quad \text{(A) } \text{ const. } \text{ flux: }\nonumber \\&\quad \left. \bar{\varvec{j}}\cdot \varvec{n}\right| _S =- \left. \bar{\varvec{\nabla }} {\bar{c}}\cdot \varvec{n} \right| _S =1, \end{aligned}$$

9g $$\begin{aligned}&\text{(B) } \text{ const. } \text{ conc.: }\nonumber \\&\quad \left. {\bar{c}} \right| _S = 1 \end{aligned}$$

with the dimensionless Peclet number

10 $$\begin{aligned} \text{(A) } \text{ const. } \text{ flux: }&\mathrm{Pe}&\equiv \mathrm{Pe}_A \equiv \frac{\kappa \alpha a^2}{D^2 \mu }= \frac{\kappa {\dot{m}}}{2\pi D^2 \mu }, \nonumber \\ \text{(B) } \text{ const. } \text{ conc.: }&\mathrm{Pe}&\equiv \mathrm{Pe}_B\equiv \frac{\kappa c_S a}{D \mu } , \end{aligned}$$

where ${\dot{m}}= 2\pi a^2\alpha$ is the mass loss per time of the swimmer. We also introduced the dimensionless Biot number

11 $$\begin{aligned} {\bar{k}} \equiv \frac{ak}{D} \end{aligned}$$

governing possible evaporation. From Eq. (9d), we see that the Peclet number $\mathrm{Pe}$ determines the velocity scale of the Marangoni flow field. Therefore, we can also assign a Reynolds number $\mathrm{Re}_{\mathrm{M}} = {2\mathrm{Pe}}/{\mathrm{Sc}}= \mathrm{Re} \mathrm{Pe}/{\bar{U}}$ to the Marangoni flow. In the following, we will address the low Reynolds number regime implying that *both* $\mathrm{Re}\ll 1$ *and* $\mathrm{Re}_{\mathrm{M}}\ll 1$ such that both flow contributions fulfill the Stokes equation. Via the advection with $\bar{\varvec{v}}(\varvec{\rho })+ \bar{\varvec{v}}_\mathrm{M}(\varvec{\rho })$, the concentration field $c(\varvec{\rho })$ depends both on the dimensionless velocity scale ${\bar{U}}$ of the Stokes field and the dimensionless velocity scale $\mathrm{Pe}$ of the Marangoni flow field, in general. All dimensionless parameters are summarized in Table 1.

### Marangoni forces, energy transduction and swimming condition

The half-spherical swimmer moving at velocity *U* must be force-free and is subject to three forces. First, there is the drag force, which is given by the standard Stokes drag for a half-sphere, $\varvec{F}_{\mathrm{D}} = F_{\mathrm{D}}\varvec{e}_z$. In dimensionless form using ${\bar{F}} \equiv F/{D\mu }$, this is

12 $$\begin{aligned} {\bar{F}}_{\mathrm{D}}= -3\pi a {\bar{U}}. \end{aligned}$$

Second, there is the direct Marangoni propulsion force $\varvec{F}_{\mathrm{M}} = F_{\mathrm{M}} \varvec{e}_z$ from integrating the surface stress $\varDelta \gamma (\varvec{r})= -\kappa c(\varvec{r})$ along the air–water–swimmer contact line *L* around the swimmer at $y=0$,

13 $$\begin{aligned} \frac{{\bar{F}}_{\mathrm{M}}}{{\mathrm{Pe}}}&= - \oint _L d{\bar{s}} (\varvec{e}_n\cdot \varvec{e}_z) {\bar{c}}(\varvec{\rho }) \nonumber \\&=- 2 \int _0^\pi d\theta \cos \theta {\bar{c}}(1,\theta )|_{{\bar{y}}=0}, \end{aligned}$$

in dimensionless form. For constant concentration boundary conditions (B), there is no direct Marangoni force ${\bar{F}}_{\mathrm{M}}=0$ because there are no concentration and, thus, surface tension gradients along the contact line *L* by definition.

Third, there is the Marangoni flow force $\varvec{F}_{\mathrm{M, fl}} = F_{\mathrm{M, fl}} \varvec{e}_z$, which is by definition the force transmitted by fluid stresses of the Marangoni flow onto the sphere, $F_{\mathrm{M, fl}} \equiv - \int _{S} da_i \sigma _{\mathrm{M, iz}}$. For low Reynolds numbers, we can employ the reciprocal theorem to calculate the Marangoni flow force without explicitly calculating the Marangoni flow $\varvec{v}_{\mathrm{M}}$ [37]. In “Appendix” A, we discuss the reciprocal theorem in terms of energy transduction and find the result (A.10), which states that the mutual power input by Marangoni stresses via the Stokes flow field is *completely* transduced via the Marangoni flow force onto the sphere, while the power input by Marangoni stresses via the Marangoni flow field itself is completely dissipated. This energy transduction statement (A.10) is equivalent to the result derived by Masoud and Stone [37] for the Marangoni flow force directly from the reciprocal theorem. In the rest frame of the sphere, we obtain in dimensionless form

14 $$\begin{aligned} \frac{{\bar{F}}_{\mathrm{M,fl}}}{{\mathrm{Pe}}}&= - \int _{S_{\mathrm{Int}}} d{\bar{S}} \frac{\bar{\varvec{v}}(\varvec{\rho })+{\bar{U}}\varvec{e}_z}{{\bar{U}}} \cdot \bar{\varvec{\nabla }}_S {\bar{c}}(\varvec{\rho }), \end{aligned}$$

where $\bar{\varvec{v}}(\varvec{\rho })/{\bar{U}}$ is the dimensionless Stokes flow field from (3a) and (3b) (in particular, this is independent of ${\bar{U}}$) in the sphere frame.

The total Marangoni force ${\bar{F}}_{\mathrm{M,tot}} = {\bar{F}}_{\mathrm{M}} + {\bar{F}}_{\mathrm{M,fl}}$ is obtained by using Eqs. (13) and (14) and the Gauss theorem,

15 $$\begin{aligned} \frac{{\bar{F}}_{\mathrm{M,tot}}}{{\mathrm{Pe}}}&= \int _{S_{\mathrm{Int}}} d{\bar{S}} \left( \bar{\varvec{\nabla }}_S\cdot \frac{\bar{\varvec{v}}(\varvec{\rho })}{{\bar{U}}}\right) {\bar{c}}(\varvec{\rho })\nonumber \\&=-\frac{3\pi }{4} \int _1^\infty d\rho \left( \frac{1}{\rho } - \frac{1}{\rho ^{3}} \right) {\bar{c}}_M(\rho ) \end{aligned}$$

$$\text{ with }{\bar{c}}_M(\rho ) \equiv \frac{2}{\pi } \int _0^\pi d\theta \cos \theta {\bar{c}}(\rho ,\theta )|_{y=0}$$.

The total Marangoni driving force has to be determined from the concentration field ${\bar{c}}(\rho ,\theta )$ of surfactant molecules (at the interface $y=0$). Note that $\bar{\varvec{\nabla }}_S\cdot \bar{\varvec{v}}(\varvec{\rho })$ is the two-dimensional surface divergence of the 3D fluid velocity field; therefore, $\bar{\varvec{\nabla }}_S\cdot \bar{\varvec{v}}(\varvec{\rho })\ne 0$ in general, although $\bar{\varvec{\nabla }}\cdot \bar{\varvec{v}}(\varvec{\rho })= 0$ for the 3D divergence of the stationary velocity field. The contribution from a constant velocity ${\bar{U}}\varvec{e}_z$ of the whole fluid (if all the fluid would be dragged along by the particle) exactly cancels the direct Marangoni force in (15), and the velocity $\bar{\varvec{v}}(\varvec{\rho })$ in the sphere frame determines the total force.

The sign of the Marangoni flow force ${\bar{F}}_{\mathrm{M,fl}}$ determines whether it increases or decreases the direct Marangoni force into the direction of higher surface tension:

- For anisotropic pure 2D surface diffusion without advection, ${\bar{c}}_{2D}(\rho ,\theta ) = \mathrm{const} + 2A_1 \rho ^{-1} \cos \theta + ...$ ($A_1<0$), as in Refs. [29, 37], we find ${\bar{F}}_{\mathrm{M,tot}}/\mathrm{Pe} = - \pi A_1 = \frac{1}{2}{\bar{F}}_{\mathrm{M}}/\mathrm{Pe}$, i.e., the total Marangoni force is half the direct Marangoni force if only the first $\cos \theta$-component is relevant. Here, Marangoni flow forces drag and *decrease* the direct driving force (${\bar{F}}_{\mathrm{M,fl}}<0$). This result will change as we (i) consider 3D diffusion and (ii) as symmetry breaking is only caused by advection, which can focus the concentration field and lead to higher Legendre components becoming relevant in ${\bar{c}}(\varvec{\rho })$.
- Because $\rho ^{-1} - \rho ^{-3} >0$ for $\rho >1$, the total Marangoni force is always positive for concentration profiles with ${\bar{c}}_M(\rho ) <0$, which are increasing toward the rear side. Vandadi *et al.* have shown that this can change in confinement, when the high of the fluid container becomes comparable to the sphere radius [31].
  For constant concentration boundary conditions (B), this means that ${\bar{F}}_{\mathrm{M,tot}} = {\bar{F}}_{\mathrm{M,fl}}>0$ because there is no direct Marangoni force ${\bar{F}}_{\mathrm{M}}=0$ for these boundary conditions.
  For constant flux boundary conditions (A), the Marangoni flow contribution ${\bar{F}}_{\mathrm{M,fl}}$, however, can have both signs. For ${\bar{F}}_{\mathrm{M,fl}}>0$, the flow force increases the direct Marangoni force resulting in ${\bar{F}}_{\mathrm{M,tot}}> {\bar{F}}_{\mathrm{M}}$; for ${\bar{F}}_{\mathrm{M,fl}}<0$, the flow force is directed backward and increases the drag force resulting in ${\bar{F}}_{\mathrm{M,tot}}< {\bar{F}}_{\mathrm{M}}$. As opposed to Ref. [29], we will find that both cases are possible. A backward force is found for steep radial gradients in the concentration ${\bar{c}}(\rho )$, which is the case for high velocities ${\bar{U}}\gg 1$ in the advection-dominated regime, and a forward force is found at low velocities ${\bar{U}}\ll 1$ in the diffusive regime.
- Advection leads to a tangential $\varvec{e}_\theta$-component of $\bar{\varvec{\nabla }}_S {\bar{c}}(\varvec{\rho })$ pointing from the front to the rear corresponding to an increasing surfactant concentration toward the rear, which gives rise to a forward Marangoni flow $\sim - \varvec{e}_\theta$. Accordingly, this increases the driving force (${\bar{F}}_{\mathrm{M,fl}}>0$) because $-\varvec{e}_z \cdot \bar{\varvec{\nabla }}_S {\bar{c}}(\varvec{\rho })\sim -\varvec{e}_z \cdot \varvec{e}_\theta \sim \sin \theta >0$ in Eq. (14). This effect dominates in the diffusive regime.
  A radial $\varvec{e}_r$-component of $\bar{\varvec{\nabla }}_S {\bar{c}}(\varvec{\rho })$ pointing inward corresponding to a radially decaying surfactant concentration and, on the other hand, gives rise to to radially outward Marangoni flows. Because $-\varvec{e}_z \cdot \bar{\varvec{\nabla }}_S {\bar{c}}(\varvec{\rho })\sim \varvec{e}_z \cdot \varvec{e}_r \propto \cos \theta$ in Eq. (14), this increases the direct force in the front (around $\theta =0$) but decreases it in the back (around $\theta =\pi$). Advection gives rise to bigger surfactant concentrations in the back, which lead to bigger radial concentration gradients on the rear side (in some distance from the sphere because constant flux boundary conditions assure uniform radial gradients right at the surface of the sphere). Overall, the radial Marangoni flows in the back are stronger and decrease the direct force or increase the drag (${\bar{F}}_{\mathrm{M,fl}}<0$). This effect dominates in the advective regime for constant flux boundary conditions (A) and is rather subtle, as can be seen from the fact that it is absent for the constant concentration boundary conditions (B), where ${\bar{F}}_{\mathrm{M,tot}} = {\bar{F}}_{\mathrm{M,fl}}>0$ always. Then, the constant concentration at the surface of the sphere leads to smaller radial concentration gradients on the rear side, because the concentration decay is stretched over a larger distance by advection. Then, radial Marangoni flows in the front are stronger and increase the direct force.
- The last equality in (15) shows that the effect of including the Marangoni flow contribution is that the total Marangoni forces are dominated by the concentration profile ${\bar{c}}(\rho ,\theta )$ around $\rho \sim 2$, where $\rho ^{-1} - \rho ^{-3}$ assumes its maximal value. Concentration boundary layer profiles concentrated around $\rho \approx 1$, as we will find for large swimmer velocities ${\bar{U}}> 1$ in the advection-dominated regime, give a small total Marangoni force (because $\rho ^{-1} - \rho ^{-3}\approx 2(\rho -1)$ is small), i.e., Marangoni flows decrease the direct Marangoni driving force.
- Long-range contributions as, for example, from a long advection tail can be important, even if they are limited to a small angular regime around $\theta \sim \pi$ as for high velocities. The highest total force is obtained if a long-range $-\cos \theta$-component is present in the concentration profile, as we will find for small swimmer velocities; then, Marangoni flows increase the direct Marangoni driving force. This makes a Marangoni swimmer also susceptible to disturbances in its far-field as, for example, induced by other swimmers.

These results for Marangoni forces as a function of ${\bar{U}}$ are inserted into the force balance or swimming condition

16 $$\begin{aligned} -{\bar{F}}_{\mathrm{D}} = 3\pi {\bar{U}}_{\mathrm{swim}} = {\bar{F}}_{\mathrm{M}}(\mathrm{Pe},{\bar{U}}_{\mathrm{swim}}) + {\bar{F}}_{\mathrm{M,fl}} (\mathrm{Pe},{\bar{U}}_{\mathrm{swim}}), \nonumber \\ \end{aligned}$$

in order to obtain an additional equation whose solution determines the actual swimmer velocity ${\bar{U}}={\bar{U}}_{\mathrm{swim}}$ as a function of the remaining control parameters $\mathrm{Pe}$ (“fuel” emission) and eventually ${\bar{k}}$ (evaporation).

### Control parameters and parameter regimes

The non-dimensionalization reveals that the coupled problems (i)–(iii) and the Marangoni forces depend on three dimensionless control parameters (see also Table 1): first, the prescribed dimensionless velocity of the swimmer ${\bar{U}}$; second, the Peclet number $\mathrm Pe$ characterizing the strength $\alpha$ of the surfactant emission, and third, the Biot number ${\bar{k}}$ characterizing the evaporation. A suitable Peclet number can be defined for both constant flux boundary conditions (A) and constant concentration boundary conditions (B). We also see that the Peclet number both controls the strength of the Marangoni flow via Eq. (9d) and the strength of all Marangoni forces. We note, however, that ${\bar{F}}_{\mathrm{M}}/\mathrm{Pe}$ and ${\bar{F}}_{\mathrm{M,tot}}/\mathrm{Pe}$ still depend on ${\bar{U}}$ and $\mathrm{Pe}$ via the dependence of ${\bar{c}}(\varvec{\rho })$ on these parameters.

Another important finding from non-dimensionalization is that the diffusion–advection problem (iii) with boundary conditions (i) decouples from the Marangoni flow problem (iib) for $\mathrm{Pe} \ll {\bar{U}}$ or $\mathrm{Re}_{\mathrm{M}} \ll \mathrm{Re}$, where $|\varvec{v}_{\mathrm{M}}| \ll |\varvec{v}|$, and we can neglect $\varvec{v}_{\mathrm{M}}$ in the advection term. Then, the concentration profile is only determined by a classic diffusion–advection problem for mass transfer from a sphere in Stokes flow in the case of constant concentration boundary conditions (B) [33–36], but with unusual constant flux boundary conditions for case (A). It becomes axisymmetric and only depends on ${\bar{U}}$. In this limit, the Marangoni flow field need not to be calculated in order to calculate the total Marangoni force for the swimming condition. This limit will be the starting point of several analytical calculations.

All in all, we have the following regimes for a symmetric Marangoni boat at low Reynolds numbers:

- ${\bar{U}}<1$ and $\mathrm{Pe}<1$: The concentration profile is governed by diffusion, which is slightly perturbed by advection and described by a linear response in diffusion–advection (iii) with respect to ${\bar{U}}$ and $\mathrm{Pe}$. Only the linear response in ${\bar{U}}$ is relevant for symmetry breaking; therefore, the Marangoni flow can be neglected for the swimming problem. Only for $\mathrm{Pe}\ll {\bar{U}}$, the Marangoni flow decouples from the advection problem and strict analytical analysis is possible. Swimming sets in (starting with ${\bar{U}}=0$) for a critical Peclet number $\mathrm{Pe} >\mathrm{Pe}_c$; if Marangoni flows forces are included, we find $\mathrm{Pe}_c\ll 1$ and the symmetry-breaking bifurcation takes place within this regime.
- ${\bar{U}}<1$ and $1<\mathrm{Pe} <\mathrm{Sc}$: All fluid flows are still at low Reynolds numbers, but Marangoni flows are relevant. The concentration profile is governed by symmetric Marangoni advection, which is slightly perturbed by a linear response in diffusion–advection (iii) with respect to ${\bar{U}}$, which causes symmetry breaking and swimming.
- $1<{\bar{U}}<\mathrm{Sc}$ and $\mathrm{Pe} <\mathrm{Sc}$: All fluid flows are still at low Reynolds numbers, but the concentration profile is governed by advection by the swimming flow for ${\bar{U}}>1$. Advection leads to the formation of a concentration boundary layer of width ${\bar{U}}^{-1/3}$ around the half-sphere for ${\bar{U}}>1$. Only for $\mathrm{Pe}\ll {\bar{U}}$, the Marangoni flow decouples from the advection problem and strict analytical analysis is possible. For $\mathrm{Pe}> {\bar{U}}$, Marangoni flows are relevant to advection, in principle, but the surfactant is transported away by the swimming flow field via the concentration boundary layer before it can advect to the Marangoni flow field. There are, however, Marangoni flows in the advection tail, which will become relevant then.

**Fig. 2:**
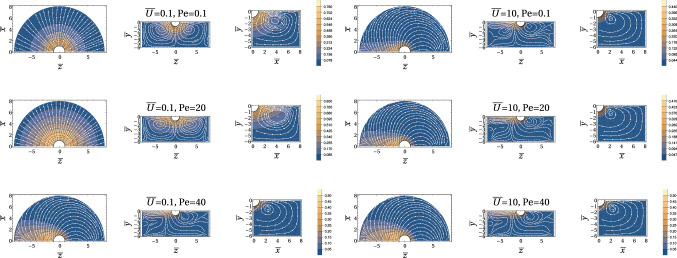
Contour plots of the concentration ${\bar{c}}(\varvec{\rho })$ and the stream lines of the Marangoni flow field $\bar{\varvec{v}}_{\mathrm{M}}(\varvec{\rho })/\mathrm{Pe}$ (in the comoving frame) for constant flux boundary conditions (A) and ${\bar{U}}=0.1,10$ and $\mathrm{Pe} = 0.1,20,40$ from numerical iterative three-dimensional FEM result for a half-cylindrical region ($0<\rho <8$, ${\bar{x}}>0$, $-6< {\bar{y}} <0$). After division by $\mathrm{Pe}$, the Marangoni flow field $\bar{\varvec{v}}_{\mathrm{M}}(\varvec{\rho })/\mathrm{Pe}$ is rather independent of $\mathrm{Pe}$ suggesting that $\bar{\varvec{v}}_{\mathrm{M}}(\varvec{\rho })/\mathrm{Pe}$ essentially depends on ${\bar{U}}$. The Marangoni flow forms a roll

Figure 2 shows exemplary numerical finite element results for the concentration field ${\bar{c}}(\varvec{\rho })$ and the stream lines of the Marangoni flow $\bar{\varvec{v}}_{\mathrm{M}}(\varvec{\rho })/\mathrm{Pe}$ for different parameter regimes for constant flux boundary conditions (A). At low velocities ${\bar{U}}\ll 1$, the Marangoni flow is mostly radial at the interface because the radial concentration profile is only slightly perturbed by advection at the interface; it features a Marangoni roll (vortex) around the swimmer with an upward flow directly around the particle. For increasing $\mathrm{Pe}$, the normalized Marangoni flow field $\bar{\varvec{v}}_{\mathrm{M}}(\varvec{\rho })/\mathrm{Pe}$ as plotted in Fig. 2 seems unchanged indicating a Marangoni flow $\bar{\varvec{v}}_{\mathrm{M}}(\varvec{\rho })$ that is simply proportional to $\mathrm{Pe}$ in strength but otherwise independent of $\mathrm{Pe}$.

At high velocities ${\bar{U}}\gg 1$, the Marangoni flow pattern changes because the concentration pattern develops the typical advection tail. As a result, there forms a vortex pair within the interface plane, which directs Marangoni flow from the tail to the front. In front of the particle, the flow reaches beneath the particle (around ${\bar{z}}=5$ in Fig. 2) and resurfaces behind the particle. This leads to a slightly distorted Marangoni vortex roll around the particle. Similar vortex patterns (with a vortex pair within the interfacial plane next to the swimmer) have been observed in Ref. [21], however, by particle image velocimetry (PIV) measurements at high Reynolds numbers. Again, for increasing $\mathrm{Pe}$, the normalized Marangoni flow field $\bar{\varvec{v}}_{\mathrm{M}}(\varvec{\rho })/\mathrm{Pe}$ seems more or less unchanged in Fig. 2.

At small ${\bar{U}}\ll 1$, the Marangoni flow force ${\bar{F}}_{\mathrm{M,fl}}>0$ will *increase* the direct Marangoni force into positive z-direction because there is a net forward tangential component of $\bar{\varvec{\nabla }}_S {\bar{c}}(\varvec{\rho })$ from the symmetry-breaking advection perturbation proportional to ${\bar{U}}$; the radial component of $\bar{\varvec{\nabla }}_S {\bar{c}}(\varvec{\rho })$ increases the drag, but is slowly decaying at small ${\bar{U}}$ and weaker.

At high ${\bar{U}}\gg 1$, on the other hand, there is a strong radial component in the concentration boundary layer around the swimmer, which increases the drag. This is created by the large radial component of $\bar{\varvec{\nabla }}_S {\bar{c}}(\varvec{\rho })$ in the concentration boundary layer region of size ${\bar{U}}^{-1/3}$ and leads to a Marangoni flow force ${\bar{F}}_{\mathrm{M,fl}}<0$ that *decreases* the direct Marangoni force. This effect is counter-intuitive as the large vortex pair suggests a strong forward Marangoni force on the large scale picture. The strong radial flows directly around the particle (which are stronger in the backward direction and, thus, dragging the particle) are not clearly visible on the larger scale in Fig. 2. The remaining total Marangoni force mainly comes from the net forward motion in the horizontal vortex pairs but will be weaker than the direct force.

### Legendre decomposition for the decoupled limit $\mathrm{Pe} \ll {\bar{U}}$

In the decoupled limit $\mathrm{Pe} \ll {\bar{U}}$, the diffusion–advection problem becomes axisymmetric. Then, ${\bar{c}}={\bar{c}}(\rho ,\theta )$ only depends on the radial coordinate and one angular coordinate, and we can also employ a decomposition of the concentration field into Legendre polynomials with respect to the angle $\theta$: ${\bar{c}}(\rho ,\theta ) = \sum _{n=0}^\infty {\bar{c}}_n(\rho ) P_n(\cos \theta )$. As derived in “Appendix” B, the diffusion–advection equation (9e) only couples coefficients ${\bar{c}}_n(\rho )$ to coefficients ${\bar{c}}_{n\pm 1}(\rho )$ because the Stokes velocity field (3a) and (3b) can be written in terms of $n=1$ polynomials only. We find the diffusion–advection equation (9e) in Legendre representation,

17 $$\begin{aligned}&\left[ \frac{1}{\rho } \partial _\rho ^2(\rho {\bar{c}}_n) - \frac{n(n+1)}{\rho ^2} {\bar{c}}_n \right] \nonumber \\&\quad = {\bar{U}} u(\rho ) \left( \frac{n}{2n-1} \partial _\rho {\bar{c}}_{n-1} + \frac{n+1}{2n+3} \partial _\rho {\bar{c}}_{n+1} \right) \nonumber \\&\qquad +{\bar{U}} \frac{v(\rho )}{\rho } \left( \frac{n(n-1)}{2n-1} {\bar{c}}_{n-1} - \frac{(n+1)(n+2)}{2n+3} {\bar{c}}_{n+1} \right) , \nonumber \\&{\bar{c}}_0(\infty ) = {\bar{c}}_\infty ~,~~ {\bar{c}}_{n>0}(\infty )=0, \nonumber \\&\text{(A) } \text{ constant } \text{ flux: }~~ \partial _\rho {\bar{c}}_0(1) = -1~,~~\partial _\rho {\bar{c}}_{n>0}(1) = 0, \nonumber \\&\text{(B) } \text{ constant } \text{ concentration: }~~ {\bar{c}}_0(1) = 1~,~~{\bar{c}}_{n>0}(1) = 0 \end{aligned}$$

for $n=0,1,....$. For small ${\bar{U}}\ll 1$, the Legendre coefficients will scale as ${\bar{c}}_n(\rho ) \sim {\bar{U}}^n$ and truncation of Legendre decomposition becomes an excellent approximation. This is one strategy for analytical progress in the linear response regime. In “Appendix” B, we also show how the Marangoni forces are expressed by the Legendre coefficients of the concentration field.

Both types of boundary conditions are completely isotropic and only $n=0$ components are nonzero. We can include traditional soap boats into our description by introducing explicitly symmetry-breaking anisotropic flux components $n>0$ into the boundary conditions, such as

18 $$\begin{aligned}&\text{(A) } \text{ constant } \text{ flux: }~~ \partial _\rho {\bar{c}}_1(1)= {\bar{\beta }} >0, \end{aligned}$$

19 $$\begin{aligned}&\text{(B) } \text{ constant } \text{ concentration: } {\bar{c}}_1(1) = {\bar{c}}_{S,1} >0 \end{aligned}$$

in the simplest generic case. Then, the soap boat emits preferentially on the lower half $\theta > \pi /2$ in case (A) or produces surfactant preferentially on the lower half of its surface in case (B) as in a standard asymmetric soap boat. Such symmetry-breaking emission will give rise to an avoided swimming bifurcation.

## Numerical methods

### Full iterative FEM solution

Numerically, we can consider the problems (i)–(iii) without further approximations at low Reynolds numbers, i.e., solve the coupled diffusion–advection problem and the Marangoni flow problem for a prescribed swimmer velocity ${\bar{U}}$.

For the coupled problems of three-dimensional coupled diffusion–advection and Marangoni flow, we use an iterative scheme of three-dimensional FEM solutions to both problems, employing FEM-routines from Wolfram MATHEMATICA in a finite cylindrical or rectangular domain. We iteratively solve for the Marangoni flow field (iib) starting from an initial guess for the concentration profile; then, we solve the diffusion–advection equation (iii) with the resulting total flow field, which gives an improved approximation for the concentration profile. With this improved approximation, we go back into solving for the Marangoni flow field (iib) and start an iteration, which should converge to the final Marangoni flow field and surfactant concentration field. The iterative approach has the advantage that the Marangoni boundary condition in the fluid flow problem (iib) is a fixed one at each iterative step and only adjusts over the iteration; the coupling of the two problems is correctly established over the iteration. Similar iterative numerical schemes for coupled problems have been applied successfully in Refs. [41, 42].

The FEM solution of the stationary equations (iib) and (iii) is obtained on a cylindrical or cubical irregular tetrahedral mesh. We use cubical (for example, with edge length 14 in ${\bar{x}}{\bar{z}}$-plane and height 7 in ${\bar{y}}$-direction in Fig. 9) or cylindrical volumes (for example, with radius 8 in ${\bar{x}}{\bar{z}}$-plane and height 4 in ${\bar{y}}$-direction in Fig. 2) for the FEM calculations. The maximal volume of mesh elements is 0.2, and the mean volume is 0.01. Mesh volumes are smaller ($<0.005$) in the region $-1<{\bar{y}}<0$ below the interface to capture Marangoni advection. Because of the mirror symmetry ${\bar{x}}\rightarrow -{\bar{x}}$, we only need to solve on half-cubes and half-cylinders ${\bar{x}}>0$ and apply Neumann boundary conditions $\left. \partial _{{\bar{x}}} {\bar{c}}\right| _{{\bar{x}}=0}=0$ and $\left. \partial _{{\bar{x}}} \bar{\varvec{v}}_{\mathrm{M}}\right| _{{\bar{x}}=0}=0$ to enforce the mirror symmetry. The boundary conditions at the outer boundaries are Dirichlet conditions for the concentration ${\bar{c}}=0$ and the Marangoni flow $\bar{\varvec{v}}_{\mathrm{M}}=0$. For sufficiently large cubes or cylinders, these boundary conditions should not matter but we still have finite size effects. In particular, at large Peclet numbers this can trigger numerical instabilities if the Marangoni roll interferes with the system boundary.

We are interested in the resulting symmetry-breaking Marangoni forces caused by a symmetry-breaking swimming motion as a function of the velocity ${\bar{U}}$. At small ${\bar{U}}$, there is the problem that artificial symmetry breaking from lattice irregularities/defects is often larger than symmetry breaking by swimming. Therefore, we average all measured quantities over two simulations with ${\bar{U}}$ and $-{\bar{U}}$ to cancel artificial symmetry-breaking effects.

### Two-dimensional FEM solution and Legendre representation for the decoupled limit $\mathrm{Pe} \ll {\bar{U}}$

For $\mathrm{Pe} \ll {\bar{U}}$, we obtain the decoupled limit, where Marangoni flow does not need to be calculated and the diffusion–advection problem becomes axisymmetric. Then, ${\bar{c}}={\bar{c}}(\rho ,\theta )$ only depends on the radial coordinate and one angular coordinate. We can solve the diffusion–advection problem in a two-dimensional angular representation using finite element methods (FEM), i.e., FEM-routines from Wolfram MATHEMATICA.

For a given ${\bar{U}}$, we can also employ the Legendre decomposition (17) of the diffusion—advection equation and calculate all functions ${\bar{c}}_n(\rho )$ by solving the resulting coupled ordinary differential equation boundary value problem. We use the MATLAB routine bvp4c for a domain $1\le \rho \le {\bar{R}}=300$ [43, 44] with Legendre components up to $n=61$. In this way, we obtain all relevant coefficients ${\bar{c}}_n(\rho )$ to calculate all Marangoni forces for the force balance.

## Diffusion–advection equation in the decoupled limit $\mathrm{Pe} \ll {\bar{U}}$ and mass transfer from a sphere in Stokes flow

First, we will consider the limit $\mathrm{Pe} \ll {\bar{U}}$, where the diffusion–advection problem for a half-sphere with prescribed velocity *U* decouples from the Marangoni flow problem because $\bar{\varvec{v}}_{\mathrm{M}}$ can be neglected. We also neglect evaporation in the beginning. This problem is axisymmetric and equivalent to mass transfer from a full sphere in laminar Stokes flow [33–36], but with unusual constant flux boundary conditions for case (A). Therefore, we first derive new analytical results for concentration profiles and for the angular dependence of the Nusselt number for these boundary conditions, both for isotropic and anisotropic emission from the sphere. In the decoupled limit, the concentration profile only depends on ${\bar{U}}$ and is independent of $\mathrm{Pe}$. Thus, the dimensionless Marangoni forces (13) and (15) only depend trivially linearly on $\mathrm{Pe}$, but ${\bar{F}}_{\mathrm{M}}/\mathrm{Pe}$ and ${\bar{F}}_{\mathrm{M,tot}}/\mathrm{Pe}$ are independent of $\mathrm{Pe}$ as well. This will make analysis of the swimming condition (16) much easier.

### Nusselt number

Diffusive release in an advecting flow can be characterized by the average Nusselt number (or Sherwood number Sh),

20 $$\begin{aligned} \mathrm{Nu} \equiv \frac{\int _S \varvec{j}(\varvec{r})\cdot \varvec{n}\,dA}{ (D/a) \int _S c(\varvec{r})\,dA} = \frac{-\partial _\rho {\bar{c}}_0(\rho =1)}{{\bar{c}}_0(\rho =1)}, \end{aligned}$$

which is the dimensionless ratio of the total emitted flux and the typical diffusive flux [36]. The average Nusselt number becomes $\mathrm{Nu}=1$ for a quiescent fluid (${\bar{U}}=0$), where the flow is purely diffusive ${\bar{c}}_0(\rho ) \propto 1/\rho$; as soon as advection is present (${\bar{U}}>0$), the current out of the sphere is increased resulting in $\mathrm{Nu}>1$. The Nusselt number thus measures how much the current out of the sphere is increased by advection over its purely diffusional value. It is an increasing function of the fluid velocity ${\bar{U}}$.

The Nusselt number has been originally defined for constant concentration boundary conditions (B), for which the result is well known [33–36],

21 $$\begin{aligned} \mathrm{Nu} = -\partial _\rho {\bar{c}}_0(\rho =1)&= {\left\{ \begin{array}{ll} 1+\frac{1}{2} {\bar{U}} + ... &{} \text{ for }~{\bar{U}}\ll 1\\ 0.6245\, {\bar{U}}^{1/3} &{} \text{ for }~{\bar{U}}\gg 1 \end{array}\right. } \end{aligned}$$

with a prefactor that can be calculated analytically [33, 36].

**Fig. 3:**
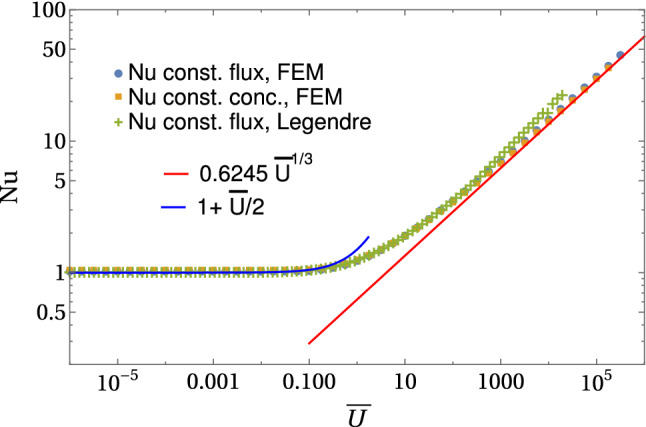
Average Nusselt number as a function of ${\bar{U}}$ for constant flux and constant concentration boundary conditions. We compare results from numerical FEM solutions of the axisymmetric diffusion–advection equation in two-dimensional angular representation with $\rho <{\bar{R}}=30$ and from numerical solutions in Legendre representation with Legendre components up to $n=61$ on a larger domain $\rho <{\bar{R}}=300$

We address the Nusselt number also for constant flux boundary conditions (A) and find a very similar result (see Fig. 3)

22 $$\begin{aligned} \mathrm{Nu} = \frac{1}{{\bar{c}}_0(\rho =1)}&= {\left\{ \begin{array}{ll} 1+\frac{1}{2} {\bar{U}} &{} \text{ for }~{\bar{U}}\ll 1\\ 0.65\, {\bar{U}}^{1/3} &{} \text{ for }~{\bar{U}}\gg 1 \end{array}\right. }, \end{aligned}$$

where the prefactor 0.65 is determined numerically from the data in Fig. 3. This result will be derived below. As opposed to the case of a constant concentration boundary condition, it is not possible to obtain an analytical result for the prefactor 0.65. Interestingly, the difference between both types of boundary conditions is small. We conclude that the Nusselt number characterizes the mass transport mechanism by the advecting fluid itself and is rather robust with respect to the emission mechanism (diffusive emission, dissolution or production by a chemical reaction at the surface) by which the transported molecules enter the advecting fluid. This is an important conclusion, which does not only apply to the microswimmer at hand, but to laminar advective mass transport phenomena in general.

**Fig. 4:**
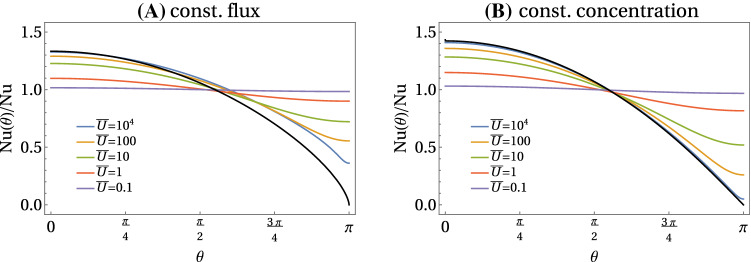
Normalized local Nusselt number $\mathrm{Nu}(\theta )/\mathrm{Nu}$ (see text) as a function of $\theta$ for constant flux (left) and constant concentration (right) boundary conditions. Colored results are from numerical FEM solutions of the axisymmetric diffusion–advection equation in two-dimensional angular representation with $\rho <{\bar{R}}=30$. Black lines are the exact analytical result (34) and the approximate analytical result (35)

**Fig. 5:**
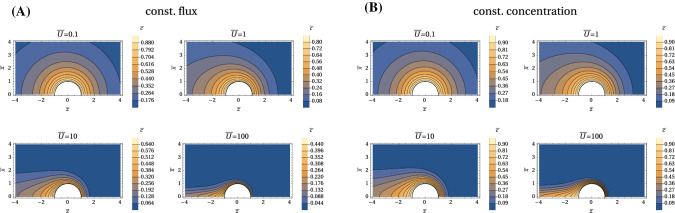
Concentration profiles in the ${\bar{z}}{\bar{x}}$-plane for (A) constant flux and (B) constant concentration boundary conditions

We can also define a local, i.e., angularly resolved Nusselt number via

23 $$\begin{aligned}&\mathrm{Nu}(\theta ) = \frac{-D\partial _r c(r=a,\theta )}{Dc(r=a,\theta )/a} = \frac{-\partial _\rho {\bar{c}}(\rho =1,\theta )}{{\bar{c}}(\rho =1,\theta )} \nonumber \\&\qquad \ \ = -\partial _\rho (\ln {\bar{c}})(\rho =1,\theta ), \nonumber \\&\text{(A) } \text{ constant } \text{ flux: }~~ \mathrm{Nu}(\theta ) = \frac{1}{{\bar{c}}(\rho =1,\theta )}, \nonumber \\&\text{(B) } \text{ constant } \text{ conc.: }~~ \mathrm{Nu}(\theta ) ={-\partial _\rho {\bar{c}}(\rho =1,\theta )}, \end{aligned}$$

which is related to the average Nusselt number by $\mathrm{Nu} = (\int _S\mathrm{Nu}(\theta )\,dA)/A_S$ for constant concentration boundary conditions (B) and $\mathrm{Nu}^{-1} = (\int _S \mathrm{Nu}^{-1}(\theta )\,dA) /A_S$ for constant flux boundary conditions (A). The local Nusselt number characterizes the symmetry breaking by advection; $\mathrm{Nu}^{-1}(\theta )$ gives the concentration profile for constant flux (A), while $\mathrm{Nu}(\theta )$ gives the emission profile for constant concentration (B). Because $\mathrm{Nu}$ and $\mathrm{Nu}(\theta )$ are still ${\bar{U}}$-dependent (see Eqs. (22) and (21)), the angular dependence in the Nusselt number profiles become more clear in the normalized local Nusselt number $\mathrm{Nu}(\theta )/\mathrm{Nu}$, which is shown for both types of boundary conditions in Fig. 4. Again, the differences between constant flux (A) and constant concentration boundary conditions (B) are surprisingly small, at least for $\theta <\pi /2$. This becomes also evident by comparing the snapshots of concentration profiles for constant concentration (B) and for constant flux boundary conditions (A) in Fig. 5.

### Main results for Marangoni forces

**Fig. 6:**
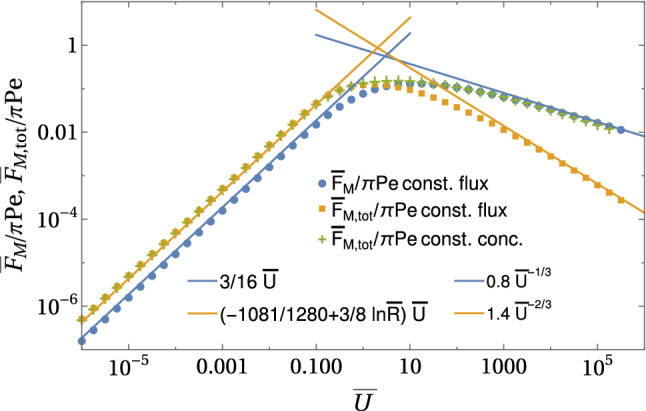
Marangoni forces ${{\bar{F}}_{\mathrm{M}}}/{\pi \mathrm Pe}$ and ${{\bar{F}}_{\mathrm{M, tot}}}/{\pi \mathrm Pe}$ for constant flux boundary conditions and ${{\bar{F}}_{\mathrm{M, tot}}}/{\pi \mathrm Pe}$ for constant concentration boundary conditions as a function of ${\bar{U}}$ in the decoupled limit $\mathrm{Pe} \ll {\bar{U}}$. All results are from numerical FEM solutions of the axisymmetric diffusion–advection equation in two-dimensional angular representation with $\rho <{\bar{R}}=30$

For constant flux boundary conditions (A), the main results for the Marangoni forces as a function of a prescribed velocity ${\bar{U}}$ are

24 $$\begin{aligned} \frac{{\bar{F}}_{\mathrm{M}} }{\pi \mathrm{Pe}}&= {\left\{ \begin{array}{ll} \frac{3}{16} {\bar{U}} &{} \text{ for }~{\bar{U}}\ll 1\\ d_{\mathrm{M}} {\bar{U}}^{-1/3}~\text{ with }~d_{\mathrm{M}}\simeq 0.8 &{} \text{ for }~{\bar{U}}\gg 1 \end{array}\right. }, \end{aligned}$$

25 $$\begin{aligned} \frac{{\bar{F}}_{\mathrm{M, tot}}}{\pi \mathrm Pe}&= {\left\{ \begin{array}{ll} - \frac{1081}{1280} {\bar{U}} +\frac{3}{8} {\bar{U}}\ln {\bar{R}} &{} \text{ for }~{\bar{U}}\ll 1\\ d_{\mathrm{M,fl}} {\bar{U}}^{-2/3}~\text{ with }~d_{\mathrm{M,fl}}\simeq 1.4 &{} \text{ for }~{\bar{U}}\gg 1 \end{array}\right. }, \end{aligned}$$

where numerical constants $d_{\mathrm{M}}$ and $d_{\mathrm{M, tot}}$ are obtained from the numerical results, see Fig. 6.

For constant concentration boundary conditions (B), there is no direct Marangoni force ${\bar{F}}_{\mathrm{M}}=0$ by definition because there are no concentration and, thus, surface tension gradients along the contact line *L*. Then, the total Marangoni force equals the Marangoni flow force and is given by

26 $$\begin{aligned} \frac{{\bar{F}}_{\mathrm{M,tot}}}{\mathrm{Pe}}&= {\left\{ \begin{array}{ll} - \frac{563}{320} {\bar{U}} +\frac{3}{8} {\bar{U}}\ln {\bar{R}} &{} \text{ for }~{\bar{U}}\ll 1\\ d_{\mathrm{M,B}} {\bar{U}}^{-1/3}~\text{ with }~d_{\mathrm{M,B}}\simeq 0.8 &{} \text{ for }~{\bar{U}}\gg 1 \end{array}\right. }, \end{aligned}$$

where the numerical constant $d_{\mathrm{M,B}}$ is obtained from the numerical results. Numerical results for these boundary conditions are also shown in Fig. 6.

The numerical result in Fig. 6 clearly confirms the existence of just two regimes for both types of boundary conditions. At small ${\bar{U}}\ll 1$, the Marangoni forces are linear in ${\bar{U}}$ for both types of boundary conditions and can be calculated as linear response in a perturbative approach. In this limit, diffusion dominates. For ${\bar{U}}\gg 1$, on the other hand, advection dominates, and a concentration boundary layer forms around the half-sphere. There is a markedly different scaling for the total Marangoni force comparing both types of boundary conditions, which we will explain below. Figure 6 shows that direct and total Marangoni force reach maximal values ${\bar{F}}_{\mathrm{M}},{\bar{F}}_{\mathrm{M, tot}}\sim 0.15\,\pi \mathrm{Pe}$ in the crossover region ${\bar{U}} \sim 1$ between diffusive and advective transport.

### Small velocity ${\bar{U}}$, perturbation theory

At small ${\bar{U}}\ll 1$, there is a linear response of the concentration field, which leads to a linear response of the Nusselt number and Marangoni forces. The coefficients can be calculated by perturbation theory about the concentration field ${\bar{c}}^{(0)}(\varvec{\rho }) = 1/\rho$ at ${\bar{U}}=0$ in powers of ${\bar{U}}$. A first approach is a naive perturbation series Ansatz

27 $$\begin{aligned} {\bar{c}}_n(\rho ) = \sum _{m=0}^\infty {\bar{U}}^m {\bar{c}}_n^{(m)}(\rho ) \end{aligned}$$

for each Legendre coefficient starting with ${\bar{c}}_0^{(0)}(\varvec{\rho }) = 1/\rho$ and ${\bar{c}}_{n>0}^{(0)}(\varvec{\rho }) = 0$. It turns out that this will work only in the “inner region” $\rho < 1/{\bar{U}}$ of a solution, because in the “outer region” $\rho \gg 1/{\bar{U}}$, the convection term can no longer be treated perturbatively, regardless how small ${\bar{U}}$ is [34]. The problem that arises in performing such a naive expansion is that already ${\bar{c}}_1^{(1)}(\rho )$ does not vanish at infinity as required by the boundary conditions. What can be done, however, is to treat a finite system $\rho < {\bar{R}}$ and apply the boundary conditions ${\bar{c}}_0({\bar{R}}) = 0$ and ${\bar{c}}_{n>0}({\bar{R}})=0$ as in the numerical approach. The above results (24), (25) and (26) are obtained by this approach. We find excellent agreement between numerics and naive perturbation theory for such finite systems.

In an infinite system, the situation differs because in the “outer region” $\rho \gg 1/{\bar{U}}$ the convection term can no longer be treated perturbatively [34]. These effects will only occur for system sizes ${\bar{R}}\gg 1/{\bar{U}}$, which become extremely large in the perturbative regime ${\bar{U}}\rightarrow 0$ of interest. To address this problem, in Ref. [34], a systematic expansion in inner and outer region and a matching procedure were performed for the constant concentration boundary condition (B), which is posed in typical heat and mass transport problems in laminar flow [33, 34, 36]. For the constant flux boundary condition (A), such calculations do not exist at the moment. We also adapt this more advanced matching procedure to the constant flux boundary condition (A). In “Appendix” C, we present the details of the perturbative approach, both the naive perturbation theory and the matching procedure. We find that in linear order in ${\bar{U}}$, both approaches still agree in the inner region. For the total Marangoni force, there is a contribution $\propto {\bar{U}}\ln {\bar{R}}$ stemming from a $\rho$-integration of a $\rho$-independent contribution to ${\bar{c}}_1^{(1)}(\rho )$ in naive perturbation theory, see Eq. (25), and (26). In the framework of the matching procedure, this contribution becomes $\propto -{\bar{U}}\ln {\bar{U}}$ as matching provides an upper cutoff ${\bar{R}}\sim 1/{\bar{U}}$ to the otherwise unchanged inner region.

Regardless of whether this contribution is regularized by system size ${\bar{R}}$ or by the boundary $\rho \sim 1/{\bar{U}}$ of the inner region, the log-divergence of this linear contribution in the total Marangoni force is a remarkable result of these calculations. Because the linear term for the direct Marangoni force stays finite, its existence means that the Marangoni flow forces strongly *increase* the direct force for ${\bar{U}}\ll 1$.

### Large velocity ${\bar{U}}$, concentration boundary layer

#### Scaling arguments

For large ${\bar{U}}\gg 1$, advection is strong and a concentration boundary layer of width $\varDelta r$ develops around the half-sphere. The width $\varDelta r$ is determined by the distance that a surfactant molecule can diffuse during the time $\varDelta t\sim a/v(\varDelta r/a)$ (see Eq. (3b)) that it takes to be transported along the sphere by advection: $\varDelta r^2 \sim D \varDelta t$. Because $v(\varDelta r/a) \sim U \varDelta r/a$ for $\varDelta r/a \ll 1$ because of the no-slip boundary condition (see Eq. (3b)), we find

28 $$\begin{aligned} \varDelta \rho = \varDelta r/a \sim {\bar{U}}^{-1/3}. \end{aligned}$$

This is a classic result for the diffusion–advection problem for constant concentration boundary conditions [33, 36], but also holds for constant flux boundary conditions.

Because the concentration will drop within the concentration boundary layer from its surface value to zero, we also have $-\partial _\rho {\bar{c}}(\rho =1,\theta ) \sim {\bar{c}}(\rho =1,\theta )/\varDelta \rho$. For constant flux boundary conditions (A) with $1= -\partial _\rho {\bar{c}}(\rho =1)$, this leads to a scaling

29 $$\begin{aligned} \mathrm{Nu}^{-1}(\theta )=\frac{1}{{\bar{c}}(\rho =1,\theta )} \sim \varDelta \rho \sim {\bar{U}}^{-1/3}~~\text{ const. } \text{ flux } \text{(A) }\nonumber \\ \end{aligned}$$

of the Nusselt number and the symmetry-breaking concentration level at the sphere. These scaling properties directly explain the results (22), $\mathrm{Nu} \sim {\bar{U}}^{1/3}$, for the Nusselt number and (24), ${\bar{F}}_{\mathrm{M}}/\mathrm{Pe}\sim {\bar{c}}(\rho =1,\theta ) \sim {\bar{U}}^{-1/3}$, for the direct Marangoni force in the limit ${\bar{U}}\gg 1$.

The result for the total Marangoni force (25) with constant flux boundary conditions deviates from this scaling. Here, the expected boundary layer scaling is ${\bar{F}}_{\mathrm{M,tot}}/\mathrm{Pe}\sim \varDelta \rho ^2 {\bar{c}}(\rho =1) \sim {\bar{U}}^{-1}$ (see Eq. (15)); this contribution is, however, only sub-dominant. The leading contribution comes from the advective tail in this limit of angular width $\varDelta \theta \sim {\bar{U}}^{1/3}$, as follows from inspection of the stream function. The (dimensionless) stream function for a sphere in Stokes flow is ${\bar{\psi }} = ({\bar{U}}/2) ( \rho ^2 + 1/2\rho - 3\rho /2) \sin ^2\theta$; in the advection-dominated regime ${\bar{U}}\gg 1$ fluid particles move along stream lines $\psi =\mathrm{const}$, and the fluid particles emerging from the boundary layer of width $\varDelta \rho \sim {\bar{U}}^{-1/3}$ around the sphere are transported into the advective tail of angular width $\varDelta \theta$ along a stream line. Therefore, $\varDelta \theta$ follows by equating the respective scaling forms of the stream function ${\bar{\psi }} \propto \rho ^2\varDelta \theta ^2$ in the tail and ${\bar{\psi }} \propto 3\varDelta \rho ^2\sin ^2\theta /2$ in the boundary layer, which gives $\rho \varDelta \theta \sim \varDelta \rho \sim {\bar{U}}^{-1/3}$. Therefore, the dominant contributions in Eq. (15) are ${\bar{F}}_{\mathrm{M,tot}}\sim \mathrm{Pe} \varDelta \theta {\bar{c}}(\rho =1,\theta ) \sim {\bar{U}}^{-2/3}$ in agreement with the numerical results in Fig. 6. This also means that the Marangoni flow forces strongly *decrease* the direct force (or effectively increase the drag) for ${\bar{U}}\gg 1$.

For constant concentration boundary conditions (B), the drop of the concentration within the boundary layer from its surface value ${\bar{c}}(\rho =1)=1$ to zero means that

30 $$\begin{aligned} \mathrm{Nu}(\theta )= -\partial _\rho {\bar{c}}(\rho =1,\theta )\sim \frac{1}{\varDelta \rho } \sim {\bar{U}}^{1/3}~~\text{ const. } \text{ conc. } \text{(B) }\nonumber \\ \end{aligned}$$

Again, these scaling properties directly explain the results (22), $\mathrm{Nu} \sim {\bar{U}}^{1/3}$, for the Nusselt number in the limit ${\bar{U}}\gg 1$. The total Marangoni force should scale ${\bar{F}}_{\mathrm{M,tot}}/\mathrm{Pe}\sim \varDelta \rho ^2 {\bar{c}}(\rho =1) \sim {\bar{U}}^{-2/3}$ from the boundary layer contribution (see Eq. (15)), which is again only subdominant. As for constant flux boundary conditions, the dominant contribution comes from the tail with ${\bar{F}}_{\mathrm{M,tot}}/\mathrm{Pe}\sim \varDelta \theta {\bar{c}}(\rho =1) \sim {\bar{U}}^{-1/3}$ which is in agreement with (26).

We also stress that, for both types of boundary conditions, we find

31 $$\begin{aligned} \mathrm{Nu}(\theta ) \sim \frac{1}{\varDelta \rho (\theta )}, \end{aligned}$$

i.e., the local Nusselt number can be interpreted as the inverse local boundary layer width, which is also evident from its definition (23) as an inverse decay length if the concentration profile drops exponentially as a function of $\rho$.

#### Rescaling and similarity transformation

More stringent arguments are based on a corresponding scale transformation of the entire diffusion–advection equation (9e) in the decoupled limit $\varvec{v}_{\mathrm{M}}\approx 0$. Expecting a boundary layer of thickness $\varDelta \rho \ll 1$, we can expand (3a) and (3b) to obtain $v(\rho ) \approx 3(\rho -1)/2$ and $u(\rho )\approx -3(\rho -1)^2/2$ to leading order. Then, we expand around the surface of the sphere $\rho =1$ by introducing a rescaled distance $\xi \equiv (\rho -1) {\bar{U}}^m$. For ${\bar{U}}\gg 1$, the leading diffusion term is radial diffusion, which scales as ${\bar{U}}^{2m}$, while the advection term scales as ${\bar{U}}^{1-m}$. If advection and diffusion are both retained in the boundary layer solution $m=1/3$ follows, which implies a boundary layer $\rho -1 \sim {\bar{U}}^{-1/3}$ as in Eq. (28). If we also scale ${\tilde{c}} \equiv {\bar{c}} {\bar{U}}^{1/3}$, the constant flux boundary condition (A) ${\bar{U}}^{1/3} \partial _\xi {\bar{c}}_0(0) = -1$ becomes ${\bar{U}}$-independent again, and we end up with ${\bar{U}}$-independent leading-order equations in the rescaled variables $\xi$ and ${\tilde{c}}$, For constant concentration boundary conditions (B), no additional rescaling of ${\bar{c}}$ is necessary, ${\bar{c}} = {\tilde{c}}$.

We obtain in the rescaled variables for ${\tilde{c}}={\tilde{c}}(\xi ,\theta )$

32 $$\begin{aligned} \partial _\xi ^2 {\tilde{c}}&= - \frac{3}{2} \xi ^2 \cos \theta \partial _\xi {\tilde{c}} + \frac{3}{2}\xi \sin \theta \partial _\theta {\tilde{c}} \nonumber \\&= -\frac{1}{2} \xi ^2 A'(\eta ) \partial _\xi {\tilde{c}} + \xi A(\eta ) \partial _\eta {\tilde{c}} \nonumber \\&~\text{ with }~~~A(\eta ) \equiv -\frac{3}{2} (1-\eta ^2), ~~\eta \equiv \cos \theta \nonumber \\&{\tilde{c}}(\infty ,\theta ) = 0, \nonumber \\&\text{(A) } \text{ constant } \text{ flux: }~~ \partial _\xi {\tilde{c}}(0,\theta ) = -1, \nonumber \\&\text{(B) } \text{ constant } \text{ conc.: }~~ {\tilde{c}}(0,\theta ) = 1, \end{aligned}$$

i.e., a parameter-free equation confirming all boundary layer scaling results (28), (29) and (30).

For the constant concentration boundary condition (B), the equations (32) can actually be solved analytically by a similarity transformation [33, 36], i.e., with an Ansatz ${\tilde{c}}(\xi ,\theta ) = f(\xi g(\cos \theta ))$ because this boundary condition is compatible to a boundary condition $f(0)=1$ for the function *f*(*x*). Exact results can be obtained for the functions $g(\eta )$ and *f*(*x*). An immediate consequence of the existence of such a solution is that the local Nusselt number is the inverse of the function $g(\cos \theta )$, and that $g(\cos \theta )$ is identical to the boundary layer width at angle $\theta$ because the function $f(\eta )$ is exponentially decaying on a scale of order unity,

33 $$\begin{aligned} \mathrm{Nu}(\theta ) = \frac{1}{g(\cos (\theta ))} = \frac{1}{\varDelta \rho (\theta )}. \end{aligned}$$

This confirms the scaling (30) and (31). The exact results for the functions $g(\eta )$ and *f*(*x*) also give the exact asymptotics of the Nusselt number in Eq. (22), $\mathrm{Nu} = -\partial _\rho {\bar{c}}_0(\xi =0) \sim c_0 {\bar{U}}^{1/3}$ with $c_0 = 3^{5/3}\pi ^{2/3}/8\varGamma (1/3) \simeq 0.624572$ [34, 36].

A similarity transformation is, however, not possible for the constant flux boundary conditions (A) $\partial _\xi {\tilde{c}}(0,\theta ) = -1$, which is incompatible with the similarity Ansatz ${\tilde{c}}(\xi ,\cos \theta ) = f(\xi g(\cos \theta ))$. It turns out that we can reformulate the results for constant concentration boundary conditions in terms of a flux balance argument, which can also apply to the constant flux boundary conditions in order to obtain an approximative result for the local Nusselt number.

#### Flux balance argument for local Nusselt number

Here, we consider the balance of the diffusive flux out of the sphere at $\rho =1$ with the advective flux assuming that a boundary layer $\varDelta \rho \ll 1$ exists to which the advective flux is constrained. We also assume that by its definition (23), the local Nusselt number can be interpreted as an inverse decay length, which is to be identified with the boundary layer width $\mathrm{Nu}(\theta ) \sim {1}/{\varDelta \rho (\theta )}$, see Eq. (31).

For the flux balance, we consider a volume from $\theta =0$ up to an angle $\theta$ around the sphere $\rho =1$. The diffusive outflux from the sphere gives the particle influx into this volume. For ${\bar{U}}\gg 1$, outflux from this volume is dominated by advection in $\theta$-direction, which is limited to the boundary layer of thickness $\varDelta \rho (\theta )=\mathrm{Nu}^{-1}(\theta )$. Both influx and outflux have to balance in a stationary state. In order to show the flux balance explicitly, we integrate on both sides of equation (32) (for the unrescaled ${\bar{c}}$ rather than ${\tilde{c}}$). The integrated diffusive term on the left hand side gives the diffusive influx

$$\begin{aligned} I_{\mathrm{in}}&= 2\pi \int _{0}^{\infty } d\xi \int _\eta ^1 d{\tilde{\eta }} \partial _\xi ^2 {\bar{c}} = - 2\pi \int _\eta ^1 d{\tilde{\eta }} \partial _\xi {\bar{c}}(\xi =0,\eta )\\&= 2\pi \int _0^\theta d{\tilde{\theta }}\sin {\tilde{\theta }} \mathrm{Nu}(\theta ) {\bar{c}}(\xi =0,\theta ). \end{aligned}$$

The integrated advective term on the right-hand side gives the advective outflux

$$\begin{aligned} I_{\mathrm{out}}&= {\bar{U}} 2\pi \int _{0}^\infty d\xi {\bar{c}}(\xi ,\eta ) \frac{3}{2} \xi (1-\eta ^2)\\&\sim 2\pi {\bar{U}} \sin ^2\theta \frac{3}{2} \mathrm{Nu}^{-2}(\theta ) {\bar{c}}(\xi =0,\theta ), \end{aligned}$$

where we used that outflux is confined to a boundary layer of size $\varDelta \rho (\theta ) = \mathrm{Nu}^{-1}(\theta )$, see Eq. (31), in the last equality. The integrated Eq. (32) thus transforms into the flux balance $I_{\mathrm{in}}(\theta )=I_{\mathrm{out}}(\theta )$.

For constant concentration boundary conditions (B), we obtain after differentiating with respect to $\theta$

$$\begin{aligned} \frac{1}{3\mathrm{const}{\bar{U}}}&= \cos \theta \frac{1}{\mathrm{Nu}^3(\theta )} -\sin \theta \frac{\mathrm{Nu}'(\theta )}{\mathrm{Nu}^4(\theta )}. \end{aligned}$$

Apart from the undetermined constant, this is exactly the differential equation governing the scaling function $g(\cos \theta )$ in the similarity solution [36], which confirms $\mathrm{Nu}(\theta ) = g(\cos \theta )$. The differential equation can be solved to give the well-known exact result [36]

34 $$\begin{aligned} \mathrm{Nu}(\theta )&= \left( 2\mathrm{const}{\bar{U}}\right) ^{1/3} \frac{\sin \theta }{(\theta -\frac{1}{2} \sin (2\theta ))^{1/3}}. \end{aligned}$$

For constant flux boundary conditions (A), $-\partial _\xi {\bar{c}}(\xi =0,\theta )=1$, we have $\mathrm{Nu}(\theta ) = 1/{\bar{c}}(\xi =0,\theta )$ and flux balance gives

$$\begin{aligned} \int _0^\theta d{\tilde{\theta }}\sin {\tilde{\theta }}&=\mathrm{const} {\bar{U}} \sin ^2\theta \frac{3}{2} \mathrm{Nu}^{-3}(\theta ). \end{aligned}$$

This can be directly integrated to give a new approximative result for the angular dependence of the Nusselt number,

35 $$\begin{aligned} \mathrm{Nu}(\theta )&= \mathrm{const} {\bar{U}}^{1/3} \left( 1+\cos \theta \right) ^{1/3}. \end{aligned}$$

The normalized local Nusselt numbers $\mathrm{Nu}(\theta )/\mathrm{Nu}$ are plotted as black lines in Fig. 4. The agreement for large ${\bar{U}}$ is excellent for constant concentration boundary conditions (B) and approximate for constant flux boundary conditions (A), as expected. The flux balance approach also confirms the scaling $\mathrm{Nu}(\theta ) \sim {\bar{U}}^{1/3}$, see Eqs. (29) and (30).

### Anisotropic emission

**Fig. 7:**
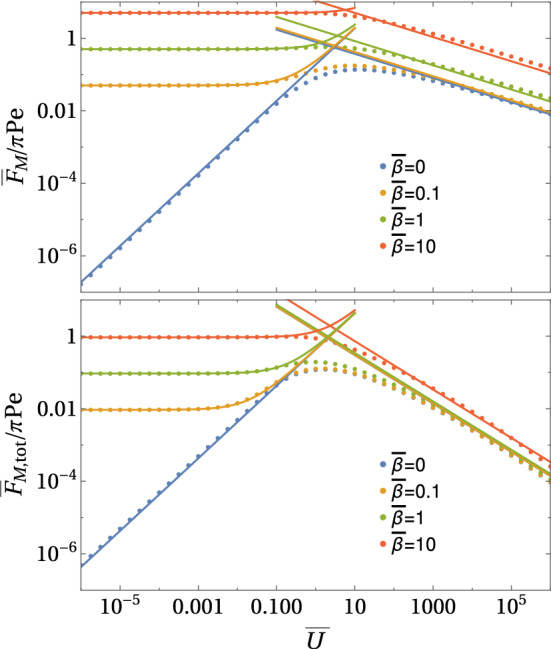
Marangoni forces ${{\bar{F}}_{\mathrm{M}}}/{\pi \mathrm Pe}$ and ${{\bar{F}}_{\mathrm{M, tot}}}/{\pi \mathrm Pe}$ for constant flux boundary conditions as a function of ${\bar{U}}$ in the presence of an anisotropy ${\bar{\beta }}$ in the emission. All results are from numerical FEM solutions of the axisymmetric diffusion–advection equation in two-dimensional angular representation with $\rho <{\bar{R}}=30$. The solid lines for ${\bar{U}}<1$ are the analytical perturbative results (36) and (37). The solid lines for ${\bar{U}}>1$ are the scaling results (38) and (39). For ${\bar{\beta }}=0$, we recover the results from Fig. 6

Finally, we want to discuss the effect of an anisotropic emission boundary condition using the example of an anisotropic diffusive flux as characterized by a parameter ${\bar{\beta }}>0$ in Eq. (18). In general, we expect higher Marangoni forces, because these forces are caused by anisotropies in the concentration profile around the half-sphere. If anisotropies are present without the need to create them by advection, this increases the Marangoni forces as can also be seen in the numerical results in Fig. 7. These numerical results also show that increasing the anisotropic emission parameter ${\bar{\beta }}$ beyond ${\bar{\beta }}\sim 1$ erases the maximum in the Marangoni forces in the crossover region ${\bar{U}} \sim 1$ between diffusive and advective transport.

In the diffusive limit ${\bar{U}}\ll 1$, the anisotropy leads to an additional zeroth-order term ${\bar{c}}_1^{(0)}(\rho )= -{\bar{\beta }}/{2\rho ^2}$ in the concentration field, which results in

36 $$\begin{aligned}&\frac{{\bar{F}}_{\mathrm{M}} }{\pi \mathrm{Pe}} \approx \frac{3}{16} {\bar{U}} + \frac{1}{2}{\bar{\beta }}, \end{aligned}$$

37 $$\begin{aligned}&\frac{{\bar{F}}_{\mathrm{M, tot}}}{\pi \mathrm Pe} \approx - \frac{1081}{1280} {\bar{U}} +\frac{3}{8} {\bar{U}}\ln {\bar{R}} + \frac{3}{32} {\bar{\beta }}, \end{aligned}$$

as derived in Appendix C (see Eqs. (C.5) and (C.6)). These perturbative results are in excellent agreement with numerical FEM results as can be seen in Fig. 7. For sufficiently small ${\bar{U}}$, the zeroth-order term dominates. If this term dominates, Marangoni flow forces *decrease* the direct force because $3 {\bar{\beta }}/32<{\bar{\beta }}/2$; this is similar to the results of Ref. [29], where also an explicitly asymmetric situation was considered.

In the advective limit ${\bar{U}}\gg 1$, a boundary layer of width $\varDelta \rho \sim {\bar{U}}^{-1/3}$ determines the physics. On the scale of the boundary layer thickness, the concentration drops from its surface value ${\bar{c}}(\rho ,\theta )$ to zero. For constant flux boundary conditions (A), this led to a concentration level ${\bar{c}}(\rho ,\theta )\sim \varDelta \rho \sim {\bar{U}}^{-1/3}$ (see Eq. (29)) at the sphere. In the presence of an explicitly symmetry-breaking emission $\partial _\rho {\bar{c}}_1(\rho =1) = {\bar{\beta }}$, this contribution will also decay on the scale of the boundary layer $\varDelta \rho$, and we expect a corresponding contribution ${\bar{\beta }} {\bar{U}}^{-1/3}$ to the concentration level at the sphere, ${\bar{c}}(\rho =1,\theta ) \sim (\mathrm{const}+{\bar{\beta }}) {\bar{U}}^{-1/3}$. Because the direct Marangoni force scales as ${\bar{F}}_{\mathrm{M}}/\mathrm{Pe}\sim {\bar{c}}(\rho =1,\theta )$, this leads to

38 $$\begin{aligned} \frac{{\bar{F}}_{\mathrm{M}} }{\pi \mathrm{Pe}}&\approx (d_{\mathrm{M}}+{\bar{\beta }}) {\bar{U}}^{-1/3}, \end{aligned}$$

which is in good agreement with numerical results as shown in Fig. 7. The total Marangoni force scaling is dominated by the advective tail, which led to ${\bar{F}}_{\mathrm{M,tot}}\sim \mathrm{Pe} \varDelta \theta {\bar{c}}(\rho =1,\theta )$; we find

39 $$\begin{aligned} \frac{{\bar{F}}_{\mathrm{M,tot}} }{\pi \mathrm{Pe}} \approx d_{\mathrm{M,\beta }} \left( \frac{d_{\mathrm{M,fl}}}{d_{\mathrm{M,\beta }}} +{\bar{\beta }}\right) {\bar{U}}^{-2/3}~\text{ with }~d_{\mathrm{M,\beta }}\simeq 0.2. \nonumber \\ \end{aligned}$$

This result is also in good agreement with numerical results as shown in Fig. 7.

## Diffusion–advection with strong Marangoni flow $\mathrm{Pe} \gg {\bar{U}}$

For a strong Marangoni flow, $\mathrm{Pe} \gg {\bar{U}}$, the linear response regime ${\bar{U}}\ll 1$ becomes modified. We first have to address the dominant Marangoni flow problem (iib), which determines the Marangoni flow $\varvec{v}_{\mathrm{M}}$. For $\mathrm{Pe} \gg {\bar{U}}$, this is the dominant contribution to the fluid flow in the diffusion–advection problem (iii). The Marangoni flow pattern is a stationary Marangoni vortex ring around the spherical swimmer below and parallel to the fluid interface $S_{\mathrm{Int}}$ as can be seen in Fig. 2. Because this solution lacks axisymmetry, a complete and analytical solution is no longer possible.

Applying mass conservation ${\bar{J}} \sim 2\pi {\bar{c}} {\bar{v}}_{\mathrm{M}}\rho {\bar{l}}_c = \mathrm{const}$ and the Marangoni boundary condition to concentration profile and Marangoni flow field in a concentration boundary layer of width ${\bar{l}}_c \sim (\rho /{\bar{v}}_{\mathrm{M}})^{1/2}$ below the fluid interface $S_{\mathrm{Int}}$, we find a scaling [25]

40 $$\begin{aligned} {\bar{c}}(\rho )&= {\bar{c}}(1) \rho ^{-2/3} ~~\text{ with }~~ {\bar{c}}(1) \sim \mathrm{Pe}^{-1/3}, \end{aligned}$$

41 $$\begin{aligned} {\bar{v}}_{\mathrm{M}}&\sim {\bar{c}}^{-2} \rho ^{-3} \sim {\bar{c}}^{-2}(1) \rho ^{-5/3} \sim \mathrm{Pe}^{2/3} \rho ^{-5/3}. \end{aligned}$$

for strong Marangoni flows. Here, we will further test this result in numerical FEM solutions,

We see that the advective current ${\bar{j}} \sim {\bar{c}} {\bar{v}}_{\mathrm{M}}\sim \mathrm{Pe}^{1/3} \rho ^{-7/3}$ becomes smaller than the corresponding diffusive current ${\bar{j}}_D \sim -\partial _\rho {\bar{c}} \sim \mathrm{Pe}^{-1/3} \rho ^{-5/3}$ for $\rho > \mathrm{Pe}$. Then, our assumption of advective transport breaks down, and this should mark the boundary of the Marangoni advection-dominated region. Therefore,

42 $$\begin{aligned} \rho _{\mathrm{M}} \sim \mathrm{Pe} \end{aligned}$$

should be the scaling of the size of the Marangoni vortex around the sphere for low Reynolds numbers. At larger distances, a crossover to diffusive transport with ${\bar{c}} \propto \rho ^{-1}$ sets in.

We can also introduce the dimensionless Marangoni number for the radial Marangoni flow, which exactly compares advective Marangoni current and diffusive current by definition,

43 $$\begin{aligned} \mathrm{Ma} = \frac{j_{\mathrm{M}}}{j} = \frac{v_{\mathrm{M}} r}{D} = {\bar{v}}_{\mathrm{M}} \rho = \mathrm{Pe}^{2/3} \rho ^{-2/3}, \end{aligned}$$

and see that $\rho _{\mathrm{M}}$ is determined by the condition that the regime $\mathrm{Ma} >1$ determines the size of the Marangoni vortex.

**Fig. 8:**
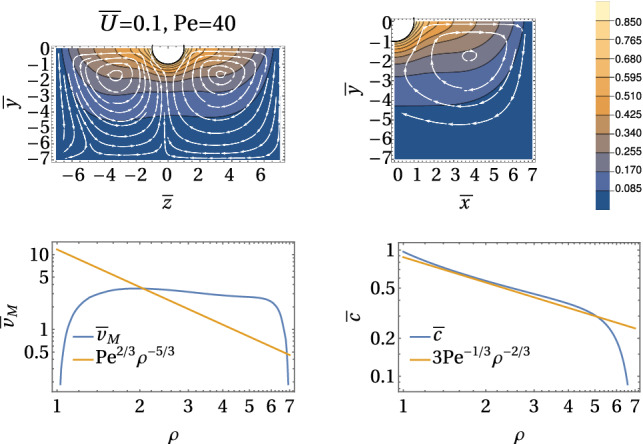
Iterative three-dimensional FEM results for $\mathrm{Pe}=40$ and ${\bar{U}}=0.1$ in a cubic system with $-7< {\bar{y}} <0$, $0<{\bar{x}}<7$, $-7<{\bar{z}}<7$. Top: Contour plots of the concentration ${\bar{c}}(\varvec{\rho })$ and the stream lines of the Marangoni flow field $\varvec{v}_{\mathrm{M}}(\varvec{\rho })/\mathrm{Pe}$. Bottom: $\varvec{v}_{\mathrm{M}}$ as a function of $\rho$ and ${\bar{c}}$ as a function of $\rho$ at the interface ${\bar{y}}=0$ along with the predictions (41) and (40)

We can test the predictions (40) and (41) in numerical FEM solutions, see Fig. 8. One problem is that, for large Peclet numbers, the finite size of the numerical system becomes too small to accommodate the Marangoni vortex of size $\rho _{\mathrm{M}} \sim \mathrm{Pe}$ properly. This results in deviations of the interfacial Marangoni flow field from Eq. (41). The numerical results for the interfacial concentration field show excellent agreement with (40).

So far, we considered the leading order of our problem by setting ${\bar{U}}\approx 0$; going one order further, we get the linear response for small ${\bar{U}}$ with the ansatz ${\bar{c}} = {\bar{c}}^{(0)} + {\bar{U}} {\bar{c}}^{(1)}$ with ${\bar{c}}^{(0)}(\rho )$ given by (40). In the total flow $\varvec{v} + \varvec{v}_{\mathrm{M}}$, the Marangoni flow (41) is the zeroth-order result, $\varvec{v}_{\mathrm{M}}= \varvec{v}_{\mathrm{M}}^{(0)}$, while the Stokes swimming flow $\varvec{v}= \varvec{v}^{(1)}$ is linear in ${\bar{U}}$. In an advection-dominated situation, mass conservation in the boundary layer still holds in the presence of Stokes flow,

$$\begin{aligned} 1 \sim ({\bar{c}}^{(0)} + {\bar{U}} {\bar{c}}^{(1)}) ({\bar{U}}{\bar{u}}\cos \theta + {\bar{v}}_{\mathrm{M}})^{1/2}\rho ^{3/2}, \end{aligned}$$

where the radial component ${\bar{u}}$ of the Stokes flow is considered. Expanding up to first order in ${\bar{U}}$, we find a scaling

$$\begin{aligned} {\bar{c}}^{(1)}(\rho ) \sim \frac{1}{ {\bar{v}}_{\mathrm{M}}^{1/2}(\rho )} {\bar{c}}^{(0)}(\rho ) {\bar{u}}(\rho ) \sim \mathrm{Pe}^{-2/3} \rho ^{1/6} {\bar{u}}(\rho ), \end{aligned}$$

which will give rise to a Marangoni force scaling

44 $$\begin{aligned} \frac{ {\bar{F}}_{\mathrm{M}}}{\pi \mathrm{Pe}}&\sim {\bar{U}} \mathrm{Pe}^{-2/3},&\frac{ {\bar{F}}_{\mathrm{M,tot}}}{\pi \mathrm{Pe}}&\sim {\bar{U}} \mathrm{Pe}^{-2/3}. \end{aligned}$$

**Fig. 9:**
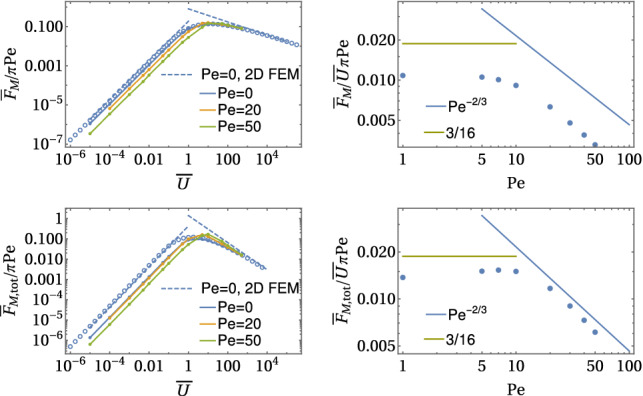
Left: Iterative three-dimensional FEM results for ${ {\bar{F}}_{\mathrm{M}}}/\pi \mathrm{Pe}$ (top) and ${{\bar{F}}_\mathrm{M,tot}}/\pi \mathrm{Pe}$ (bottom) as a function of ${\bar{U}}$ for $\mathrm{Pe} = 0-50$ for a cubic system with $-7< {\bar{y}} <0$, $0<{\bar{x}}<7$, $-7<{\bar{z}}<7$. Blue open circles are results for $\mathrm{Pe}=0$ from FEM solutions to the axisymmetric diffusion–advection equation in two-dimensional angular representation with ${\bar{R}}=30$. The slope in the linear response regime for ${\bar{U}}\ll 1$ is reduced according to Eq. (44). Results for ${\bar{U}}\gg 1$ are essentially not affected by strong Marangoni flows $\mathrm{Pe}\gg {{{\bar{U}}}}$. Right: Corresponding slopes ${ {\bar{F}}_{\mathrm{M}}}/{\bar{U}}\pi \mathrm{Pe}$ and ${{\bar{F}}_\mathrm{M,tot}}/{\bar{U}}\pi \mathrm{Pe}$ as a function of $\mathrm{Pe}$ calculated from the results for ${\bar{U}}=0.1$

Numerical FEM results show that both prefactors are of order unity (but hard to quantify because of finite size effects), see Fig. 9. This shows that Marangoni flows depress the total driving force in the linear response regime by a factor $\mathrm{Pe}^{-2/3}$ because it is harder to break the symmetry in the presence of the strong Marangoni flow advection. Numerical results in Fig. 9 also show that the total Marangoni force is somewhat larger than the direct Marangoni force, ${\bar{F}}_{\mathrm{M,tot}} > {\bar{F}}_{\mathrm{M}}$. In this respect, our previous results for linear response regime for $\mathrm{Pe}\ll {\bar{U}}$ remain unchanged: The Marangoni flow force *increases* the direct force.

In the advection-dominated regime ${\bar{U}}\gg 1$, on the other hand, results are essentially not affected by strong Marangoni flows $\mathrm{Pe}\gg {{{\bar{U}}}}$ as the numerical results in Fig. 9 show. The flow field $\varvec{v}$ will still give rise to a concentration boundary layer of thickness $\varDelta \rho \sim {\bar{U}}^{-1/3}$ around the sphere. On the scale of the boundary layer, the Marangoni flows $\varvec{v}_{\mathrm{M}}$ are not yet developed; they develop only further away at $1\ll \rho < \rho _{\mathrm{M}} \sim \mathrm{Pe}$ because of the no-slip boundary condition for the Marangoni flow in (iib). Therefore, the results for ${\bar{U}}\gg 1$ are essentially unaffected by a strong Marangoni flow for $\mathrm{Pe}\gg {{{\bar{U}}}}$.

## Diffusion–advection in the presence of evaporation

In the presence of evaporation, we have a convective (Robin) boundary condition (9b), which is governed by the dimensionless Biot number (11), instead of the Neumann condition (9a), which is recovered for vanishing Biot number ${\bar{k}}=0$. In general, evaporation of surfactant depletes the interface of surfactant and, thus, decreases the Marangoni driving forces (both direct and flow forces). For volatile camphor, we find a Biot number ${\bar{k}} = ak/D \approx 550$ using results from Ref. [18], whereas other surfactants such as PEG are non-volatile and have a very small Biot number [25].

The Biot number can also be interpreted as an extrapolation length scale. The concentration profile will fall off exponentially perpendicular to the interface in the outward direction on a dimensionless extrapolation length scale $\varDelta {\bar{y}}\sim 1/{\bar{k}}$ given by the inverse of the Biot number.

In Ref. [25], we developed a qualitative scaling theory based on the assumption that the total evaporation flux balances the total emission flux of surfactant in a stationary state. In the diffusive regime ${\bar{U}}\ll 1$, this leads to

45 $$\begin{aligned} {\bar{F}}_{\mathrm{M}}&\sim \left. {\bar{F}}_{\mathrm{M}}\right| _{{\bar{k}}=0} \frac{1}{{\bar{k}}+1},&{\bar{F}}_{\mathrm{M,tot}}&\sim \left. {\bar{F}}_{\mathrm{M,tot}}\right| _{{\bar{k}}=0} \frac{1}{{\bar{k}}+1}. \end{aligned}$$

In the advection-dominated limit ${\bar{U}}\gg 1$, we find

46 $$\begin{aligned} {\bar{F}}_{\mathrm{M}}&\sim \left. {\bar{F}}_{\mathrm{M}}\right| _{{\bar{k}}=0} \frac{{\bar{U}}^{1/3}}{{\bar{k}}+{\bar{U}}^{1/3}},&{\bar{F}}_{\mathrm{M,tot}}&\sim \left. {\bar{F}}_{\mathrm{M,tot}}\right| _{{\bar{k}}=0} \frac{{\bar{U}}^{1/3}}{{\bar{k}}+{\bar{U}}^{1/3}}. \end{aligned}$$

In both limits, Marangoni forces are reduced by evaporation, because it reduces the surfactant concentration.

## Swimming condition, symmetry breaking and speed

Now, we have a rather complete picture of the solution of problems (i)–(iii), i.e., diffusion–advection coupled to hydrodynamics for a prescribed swimmer velocity ${\bar{U}}$ at low Reynolds numbers. In particular, we know the Marangoni forces as a function of the prescribed velocity ${\bar{U}}$.

### Swimming condition

The swimming condition (16) gives an additional force balance relation between Marangoni forces and ${\bar{U}}$, which has to be satisfied in the swimming state and determines the selected swimming speed ${\bar{U}}={\bar{U}}_{\mathrm{swim}}$ as a function of Peclet number $\mathrm{Pe}$ and Biot number ${\bar{k}}$. In general, the swimming velocity increases with $\mathrm{Pe}$ and decreases with ${\bar{k}}$.

The force balance condition can be interpreted such that intersections of the linear Stokes friction relation $-{\bar{F}}_{\mathrm{D}}= 3\pi {\bar{U}}$ and the total Marangoni force ${\bar{F}}_{\mathrm{M, tot}} = {\bar{F}}_{\mathrm{M, tot}}({\bar{U}})$ relation give the swimming speed ${\bar{U}}={\bar{U}}_{\mathrm{swim}}$. The resulting swimming state can only be stable if the Marangoni force curve ${\bar{F}}_{\mathrm{M, tot}}({\bar{U}})$ intersects the straight Stokes friction line $3\pi {\bar{U}}$ from *above*. Then, a speed fluctuation $\delta {\bar{U}}>0$ will give rise to ${\bar{F}}_{\mathrm{M, tot}}<-{\bar{F}}_{\mathrm{D}}$ such that friction dominates, and the swimming speed is decreased again.

All curves $({\bar{F}}_{\mathrm{M, tot}}/\pi \mathrm{Pe})({\bar{U}})$ in Figs. 6, 7 and 9 start linearly $\propto {\bar{U}}$ in the diffusive regime ${\bar{U}}\ll 1$ and then cross over to sublinear growth and finally decrease in the advective regime ${\bar{U}}>1$. Therefore, all intersection points with the linear Stokes friction function will represent *stable* swimming states, also if an anisotropic emission is included. These results remain unchanged if evaporation is included.

**Fig. 10:**
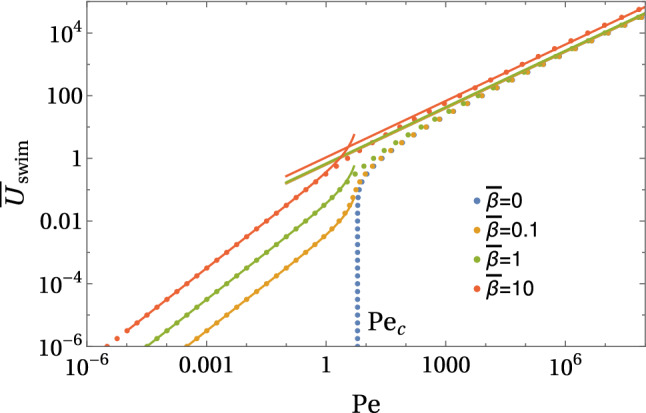
Swimming speed ${\bar{U}}_{\mathrm{swim}}$ as a function of the Peclet number $\mathrm{Pe}$ (representing emission strength) based on Marangoni forces from the FEM solutions in Fig. 7 in the absence of evaporation and in the decoupled limit. For ${\bar{\beta }}=0$, the blue vertical line of data points ends in the critical Peclet number $\mathrm{Pe}_c$ at zero swimming speed. In the presence of an anisotropic emission ${\bar{\beta }}>0$, the swimming bifurcation in the diffusive regime becomes avoided resulting in an initial linear relation ${\bar{U}}_{\mathrm{swim}}\propto \mathrm{Pe}$, crossing over to ${\bar{U}}_{\mathrm{swim}}\propto \mathrm{Pe}^{3/5}$ in the advective regime. The solid lines for ${\bar{U}}<1$ are derived from the analytical perturbative results (36) and (37). The solid lines for ${\bar{U}}>1$ are derived from the scaling results (38) and (39)

In the decoupled limit $\mathrm{Pe}\ll {\bar{U}}$, the total Marangoni force is always trivially linear in $\mathrm{Pe}$. Then, we can directly obtain the swimming condition in the form

47 $$\begin{aligned} \mathrm{Pe}&= \frac{3{\bar{U}}_{\mathrm{swim}}}{({\bar{F}}_{\mathrm{M, tot}}/\pi \mathrm{Pe})({\bar{U}}_{\mathrm{swim}})}. \end{aligned}$$

Using the Marangoni forces from Fig. 7 in the decoupled limit, which include an asymmetric emission ${\bar{\beta }}$ and reduce to Eq. (25) for ${\bar{\beta }}=0$ and inverting this relation, we obtain the swimming relation in Fig. 10.

### Swimming bifurcation

For ${\bar{\beta }}=0$, i.e., a symmetrically emitting swimmer, we see a sharp spontaneous symmetry breaking above a critical Peclet number $\mathrm{Pe}_c$ in the swimming relation in Fig. 10 (blue vertical line of data points). From Eq. (25), we obtain the existence of a symmetry-broken swimming state for $\mathrm{Pe}> \mathrm{Pe}_c \sim 8/\ln {\bar{R}} \rightarrow 0$, which approaches zero for large system sizes. Therefore, the symmetry is essentially always spontaneously broken in a large swimming vessel.

The swimming bifurcation in the force balance is governed by the leading-order linear terms $\propto {\bar{U}}$ (from the drag force and the linear response regime of the Marangoni forces) and the next order correction $\propto {\bar{U}}^3$ in the Marangoni force. Therefore, we expect a supercritical pitchfork bifurcation analogously to a $\phi ^4$-theory for a second-order phase transition. In the presence of the additional symmetry-breaking emission rate, ${\bar{\beta }}>0$, which contributes a constant ${\bar{U}}^0$-term to the force balance. This corresponds to an additional symmetry-breaking field in the $\phi ^4$-theory and gives rise to an avoided bifurcation. This bifurcation scenario is clearly reflected in Fig. 10.

Figure 10 and Eq. (25) were, however, derived for the decoupled limit $\mathrm{Pe}\ll {\bar{U}}$. At the swimming bifurcation, we have $\mathrm{Pe} =\mathrm{Pe}_c \gg {\bar{U}}\approx 0$, such that the feedback of Marangoni flows onto the diffusion–advection problem has to be taken into account, and the decoupling approximation should not be used. Then, Eq. (44) describes the Marangoni forces in the linear response regime, which further reduces the critical Peclet number to $\mathrm{Pe}_c \sim 1/(\ln {\bar{R}})^{3}\rightarrow 0$. In the presence of evaporation with ${\bar{k}}\gg 1$, as appropriate for surfactants such as camphor, the total Marangoni force is further depressed according to Eq. (45) resulting in an increased $\mathrm{Pe}_c = {\bar{k}}^3/(\ln {\bar{R}})^{3} \rightarrow 0$, which is, however, still approaching zero for large swimming vessel sizes ${\bar{R}}$.

For strong Marangoni flows and in the presence of evaporation, we still have a linear response of the Marangoni forces for small ${\bar{U}}$ (see Fig. 9 and Eq. (45)) with higher-order correction terms competing with a linearly ${\bar{U}}$-dependent drag force. Therefore, the above supercritical bifurcation scenario should persist.

### Swimming relation

For $\mathrm{Pe}> \mathrm{Pe}_c$, a spontaneously symmetry-broken swimming state with ${\bar{U}}_{\mathrm{swim}}>0$ exists for a symmetrically emitting swimmer with ${\bar{\beta }}=0$. Because the Marangoni force Eq. (25) remains approximately linear up to ${\bar{U}}\sim O(1)$, as can also be seen in Fig. 6, the swimming velocity rises steeply for $\mathrm{Pe}\gtrsim \mathrm{Pe}_c$ and quickly enters the asymptotics for the advection-dominated regime ${\bar{U}}_{\mathrm{swim}} \gg 1$ as can be clearly seen in Fig. 10.

In the advective regime, we find the swimming relations

48a $$\begin{aligned} {\bar{U}}_{\mathrm{swim}}&\sim \mathrm{Pe}^{3/5}&\text{ for }~ {\bar{k}}\ll \mathrm{Pe}^{1/5}, \end{aligned}$$

48b $$\begin{aligned} {\bar{U}}_{\mathrm{swim}}&\sim {\bar{k}}^{-3/4} \mathrm{Pe}^{3/4}&\text{ for }~ {\bar{k}}\gg \mathrm{Pe}^{1/5}. \end{aligned}$$

Also in this regime, we have $\mathrm{Pe} \gg {\bar{U}}_{\mathrm{swim}}$ such that Marangoni flows are strong, but this has little influence on the swimming speed because of the concentration boundary layer that forms in this regime. Evaporation is significant for ${\bar{k}} \gg \mathrm{Pe}^{1/5}$ and reduces the swimming speed because it reduces the driving Marangoni forces.

For an anisotropically emitting swimmer with ${\bar{\beta }}>0$, the bifurcation is avoided, and we find a linear swimming relation for small $\mathrm{Pe}$. In the vicinity of the bifurcation, the force balance can be written as ${\bar{U}}_{\mathrm{swim}} = (\mathrm{Pe}/\mathrm{Pe}_c) {\bar{U}}_{\mathrm{swim}} +\mathrm{Pe}{\bar{\beta }}/32$, which results in the linear swimming relation

49 $$\begin{aligned} {\bar{U}}_{\mathrm{swim}}&= \frac{\mathrm{Pe}}{1-\mathrm{Pe}/\mathrm{Pe}_c} \frac{{\bar{\beta }}}{32}. \end{aligned}$$

This describes the linear relations ${\bar{U}}_{\mathrm{swim}}\propto \mathrm{Pe}$ for small $\mathrm{Pe}$ in the swimming relation in Fig. 10. In the advective regime, we still have a crossover to the above swimming relations (48), but with a slightly increased prefactor, i.e.,

50a $$\begin{aligned} {\bar{U}}_{\mathrm{swim}}&\sim (\mathrm{const} + {\bar{\beta }}) \mathrm{Pe}^{3/5} , \end{aligned}$$

50b $$\begin{aligned} {\bar{U}}_{\mathrm{swim}}&\sim {\bar{k}}^{-3/4} (\mathrm{const} + {\bar{\beta }}) \mathrm{Pe}^{3/4}. \end{aligned}$$

## Discussion and conclusion

At low Reynolds numbers, we developed a complete theory for Marangoni boat propulsion for a completely symmetric, half-spherical, surfactant emitting swimmer. Symmetric PEG–alginate Marangoni surfactant boats can be produced down to radii $a\sim 150\,\mathrm{\mu m}$ [25] with Reynolds numbers $\mathrm{Re} \sim 1-10$ such that the low Reynolds number regime becomes accessible for surfactant-loaded boats. Recently, asymmetric thermal Marangoni surfers propelled by the thermal Marangoni effect were successfully realized [26]. Here, the thermal diffusion constant replaces the surfactant diffusion constant and is by a factor $O(10^3)$ larger. Moreover, radii $a \sim 3\,\mathrm{\mu m}$ could be reached. At the same time, swimming velocities are still in the range above $10^3-10^5 \, \mathrm{\mu m/s}$. These parameters correspond to dimensionless velocities ${\bar{U}}_{\mathrm{swim}} \sim 2\times 10^{-2}-2$, which is mostly in the diffusive regime ${\bar{U}}\ll 1$ and at low Reynolds numbers $\mathrm{Re} \sim 6\times 10^{-6}$. These swimmers were asymmetrically heated with a temperature difference $\varDelta T$ across the swimmer corresponding to a constant concentration asymmetry ${\bar{c}}_{S,1} \propto \varDelta T$. We therefore expect to be in a constant concentration situation, which is analogous to the linear regime in the constant flux swimming relation in Fig. 10. This is in accordance with the theoretical results of Würger [30], because advection plays no role in this regime and agrees with the experimental observations in Ref. [26].

Our theoretical description comprises the coupled problems of surface tension reduction by surfactant adsorption at the air–water interface including the possibility of surfactant evaporation, fluid flow (both Marangoni flow and flow induced by swimmer motion), diffusion and advection of the surfactant. Conceptually, there is no difference for a thermal Marangoni surfer as realized in Ref. [26]. In previous theoretical approaches to surfactant [29] or thermal [30] Marangoni boats, advection has been neglected. For surfactant driven Marangoni boats, this is typically a bad approximation as estimates in Ref. [25] show; for thermal Marangoni boats, this is typically justified as our above estimates show.

The three coupled problems of surfactant adsorption, low Reynolds number fluid flow and diffusion–advection of surfactant are first solved for prescribed swimmer velocity *U*; the actual swimming velocity $U_{\mathrm{swim}}$ is determined by force balance between the drag force, the direct Marangoni force from the surface tension contribution at the air–water–swimmer contact line and the Marangoni flow force. We employ the reciprocal theorem, which we could reinterpret in terms of energy transduction, to calculate the Marangoni forces.

Non-dimensionalization reveals that two dimensionless control parameters exist, the Peclet number (10), which is the dimensionless emission rate of surfactant, and the Biot number (11), which is the dimensionless evaporation rate. Evaporation is practically absent for PEG (Biot number ${\bar{k}}\ll 1$), but strong for other frequently studied soap boat swimmers such as camphor boats (Biot numbers ${\bar{k}}\approx 550$ [18]). In Ref. [25], it is shown that evaporation is relevant to quantitatively understand the large differences in the swimming relation ${\bar{U}}_{\mathrm{swim}}={\bar{U}}_{\mathrm{swim}}(\mathrm{Pe})$ between PEG–alginate swimmers and camphor boats from Ref. [20], but these Marangoni boats operate at moderate Reynolds numbers. For thermal Marangoni surfers [26], evaporation corresponds to a convective boundary condition for heat transfer from the water surface to the air; the corresponding convection coefficient will depend on the nature of the air flow that is applied to transfer heat, which is difficult to quantify. It also depends on the temperature difference to the surrounding air that can be established. Because the thermal Marangoni surfers from Ref. [26] mostly operate in the diffusive regime ${\bar{U}}\ll 1$, we expect convection to reduce the Marangoni force according to Eq. (45) if the corresponding Biot number ${\bar{k}}$ is sufficiently high.

Moreover, the dimensionless swimmer velocity ${\bar{U}}$ plays an important role as it controls the transition from a diffusive regime ${\bar{U}}\ll 1$ to an advective regime ${\bar{U}}\gg 1$. Non-dimensionalization of the coupled equations also shows a decoupling of the Marangoni flow problem for weak Marangoni flows $\mathrm{Pe}\ll {\bar{U}}$. Then, the concentration field around the interfacial Marangoni swimmer with velocity *U* is essentially equivalent to the concentration field around a mass emitting sphere moving with velocity *U* through a bulk viscous fluid, which is a classical diffusion–advection problem. We developed solutions for this diffusion–advection problem for two types of boundary conditions which seem most important for applications: constant flux boundary conditions (A) for diffusive emission of surfactant from the swimmer and constant concentration boundary conditions (B) if the surfactant dissolves from the surface or is produced by a chemical reaction on the surface. We could obtain novel results for constant flux boundary conditions, which are unusual in the diffusion–advection literature. In particular, we could obtain qualitative results for the local Nusselt number by a novel flux balance argument. All theoretical results are supported by numerical FEM simulations.

Apart from extensive results for the decoupled limit $\mathrm{Pe}\ll {\bar{U}}$, we also addressed strong Marangoni flow in the limit $\mathrm{Pe}\gg {\bar{U}}$ and evaporation on the basis of scaling arguments and numerical FEM simulations. This allowed us to obtain the Marangoni forces as a function of a prescribed swimmer speed ${\bar{U}}$ for all relevant situations, also including a possible anisotropic emission. For all cases, our theoretical results agree well with the numerical FEM calculations. Knowledge of the Marangoni forces is the basis to discuss the swimming bifurcation and swimming speed as a function of the Peclet number as main control parameter via the force balance condition.

We showed that a spontaneous symmetry breaking, i.e., a spontaneous transition into a swimming state, is possible also for a completely symmetric swimmer above a critical Peclet number. The swimming bifurcation is a supercritical pitchfork bifurcation analogous to a second order symmetry-breaking phase transition, and the presence of an explicitly symmetry-breaking emission gives rise to an avoided bifurcation. Spontaneous symmetry breaking resulting in propulsion is possible by establishing an asymmetric surfactant concentration profile that is maintained by advection. The symmetry breaking mechanism is similar to what has been proposed for autophoretic swimmers [9, 10] and liquid Marangoni swimmers [8] before.

In Eq. (48), we obtain the power-laws governing the swimming velocity as a function of Peclet and Biot number, which are ${\bar{U}}_{\mathrm{swim}} \propto \mathrm{Pe}^{3/5}$, without evaporation (PEG) and ${\bar{U}}_{\mathrm{swim}} \propto {\bar{k}}^{-3/4} \mathrm{Pe}^{3/4}$, in the presence of strong evaporation (camphor). In Eq. (50), the result is extended in the presence of an explicitly symmetry-breaking emission. Then, a linear regime emerges in the diffusive limit ${\bar{U}}\ll 1$, which is caused by the avoided bifurcation. This regime is observed for the thermal Marangoni surfers in Ref. [26].

## Energy transduction and reciprocal theorem

In this Appendix, we discuss the reciprocal theorem in terms of energy transduction, in order to see how power input from Marangoni stresses via the flow fields is transduced to the sphere for propulsion. This will also provide an alternative derivation of the results obtained by Masoud and Stone [37] via the reciprocal theorem.

For this, we switch to the laboratory frame in the following. We first consider the dissipation rate $\varPhi \equiv 2\mu \int _V dV e_{ij} e_{ij}$ of a solution of the Stokes equation in an arbitrary volume *V*. Here, $e_{ij} = \frac{1}{2}\left( \partial _i v_j + \partial _j v_i \right)$ is the strain tensor, and $\sigma _{ij} = -p \delta _{ij} + 2\mu e_{ij}$ is the stress tensor. The kinetic energy $K = \frac{1}{2} \rho \int _V dV \varvec{v}^2$ of the fluid changes according to $dK/dt = \int _{\partial V} da_i v_j \sigma _{ij} - \varPhi$, i.e., by the external power input $P_{\partial V}$ across the surface of the volume and by dissipation. In a stationary state, $dK/dt=0$ and dissipation and power input are equal,

A.1 $$\begin{aligned} \varPhi = P_{\partial V} = \int _{\partial V} da_i v_j \sigma _{ij} \end{aligned}$$

($d\varvec{a}$ is the outward normal to volume *V*). Applying this equality to the Stokes flow field $\varvec{v}$ and the liquid volume with the boundary $\partial V=S+S_{\mathrm{Int}}$ and using $\sigma _{ij}=0$ at the liquid–air interface $S_{\mathrm{Int}}$, we find

A.2 $$\begin{aligned} \varPhi&= \int _{S} da_i v_{j} \sigma _{ij} + \int _{S_{\mathrm{Int}}} da_i v_{j} \sigma _{ij} \nonumber \\&= P_S = U_j \int _{S} da_i \sigma _{ij} = -U F_D >0 \end{aligned}$$

with the Stokes drag force $F_{D} \equiv - \int _{S} da_i \sigma _{\mathrm{iz}}$ from Eq. (12). Applying the same dissipation relation (A.1) to the Marangoni flow field $\varvec{v}_{\mathrm{M}}$ and the liquid volume with the boundary $\partial V = S+S_{\mathrm{Int}}$ and using the no-slip condition $\varvec{v}_{\mathrm{M}}=0$ at the half-sphere *S*, we find

A.3 $$\begin{aligned} \varPhi _{\mathrm{M}}&= \int _{S_{\mathrm{Int}}} da_i v_{\mathrm{M,j}} \sigma _{M,ij} = P_{M, S_{\mathrm{Int}}}. \end{aligned}$$

This means that the power input by Marangoni stresses $\sigma _{M,ij}$ on the interface $S_{\mathrm{Int}}$ via the Marangoni flows $v_{\mathrm{M,j}}$ (right hand side) is dissipated entirely within the fluid without transmitting any mechanical power onto the half-sphere because $\varvec{v}_{\mathrm{M}}=0$ on *S*.

Now, we consider the mutual dissipation rate for *two* solutions $\varvec{v}^{(1)}(\varvec{r})$ and $\varvec{v}^{(2)}(\varvec{r})$ to the Stokes equation. The mutual dissipation can be shown to be given by the mutual power input in a stationary state,

A.4 $$\begin{aligned} \varPhi ^{(12)}&= 2\mu \int _V dV e^{(1)}_{ij} e^{(2)}_{ij} = \int _{\partial V} da_i v^{(2)}_j \sigma ^{(1)}_{ij}. \end{aligned}$$

The symmetry $\varPhi ^{(12)}=\varPhi ^{(21)}$ leads directly to the reciprocal theorem

A.5 $$\begin{aligned} \int _{\partial V} da_i v^{(2)}_j \sigma ^{(1)}_{ij} = \int _{\partial V} da_i v^{(1)}_j \sigma ^{(2)}_{ij}. \end{aligned}$$

Now, we can apply this finding to the Stokes and Marangoni flow fields, which both satisfy the Stokes equation, and to the liquid volume with the boundary $S+S_{\mathrm{Int}}$ resulting in a mutual dissipation

A.6 $$\begin{aligned} \varPhi _{\mathrm{mut}}&= \int _{S+S_{\mathrm{Int}}} da_i v_{j} \sigma _{\mathrm{M, ij}} + \int _{S+S_{\mathrm{Int}}} da_i v_{\mathrm{M,j }} \sigma _{ij} \nonumber \\&= 2\int _{S+S_{\mathrm{Int}}} da_i v_{j} \sigma _{\mathrm{M, ij}} = 2\int _{S+S_{\mathrm{Int}}} da_i v_{\mathrm{M,j }} \sigma _{ij} = 0, \end{aligned}$$

because $v_{\mathrm{M,j}} =0$ on the surface *S* of the half-sphere and $\sigma _{ij}=0$ on the interface $S_{\mathrm{Int}}$ such that the last equality holds. The reciprocal theorem is thus equivalent to a vanishing mutual dissipation between Stokes and Marangoni flow.

This has consequences for the dissipation relation for the total flow field $\varvec{v}_{\mathrm{tot}} = \varvec{v}+ \varvec{v}_{\mathrm{M}}$, which also includes the mutual dissipation $\varPhi _{\mathrm{mut}}$ of both contributions. The total power transmission onto the fluid volume with the boundary $\partial V = S+S_{\mathrm{Int}}$ is $P_{\mathrm{tot, S}} +P_{\mathrm{tot, S_\mathrm{Int}}}$ with

A.7 $$\begin{aligned} P_{\mathrm{tot, S_\mathrm{Int}}}&= \int _{S_{\mathrm{Int}}} da_i (v_j + v_{\mathrm{M,j}}) \sigma _{\mathrm{M,ij}} \nonumber \\&= \int _{S_{\mathrm{Int}}} da_i v_j\sigma _{\mathrm{M,ij}} + P_{M, S_{\mathrm{Int}}}, \nonumber \\ P_{\mathrm{tot, S}}&= \int _{S} da_i v_j (\sigma _{ij}+ \sigma _{\mathrm{M,ij}}) = P_S - UF_{\mathrm{M,fl}}, \end{aligned}$$

by employing boundary conditions on *S* and $S_{\mathrm{Int}}$, Eqs. (A.3) and (A.2), and by introducing the Marangoni flow force

A.8 $$\begin{aligned} F_{\mathrm{M, fl}} \equiv - \int _{S} da_i \sigma _{\mathrm{M, iz}} \end{aligned}$$

as the drag force exerted by the Marangoni flow field onto the sphere. ($d\varvec{a}$ is the inward normal to the sphere.) $F_{\mathrm{M, fl}} <0$ signals additional Marangoni drag, while $F_{\mathrm{M, fl}} >0$ signals an additional Marangoni flow propulsion force. Because of the vanishing mutual dissipation, we obtain for the total dissipation

A.9 $$\begin{aligned} \varPhi _{\mathrm{tot}}&= \varPhi + \varPhi _{\mathrm{M}} + 2\varPhi _{\mathrm{mut}} \nonumber \\&=\varPhi + \varPhi _{\mathrm{M}} = P_S + P_{M, S_{\mathrm{Int}}}. \end{aligned}$$

The equality $\varPhi _{\mathrm{tot}} = P_{\mathrm{tot, S}} +P_{\mathrm{tot, S_\mathrm{Int}}}$ and Eq. (A.7) finally gives the energy transduction for the fluid,

A.10 $$\begin{aligned} P_S + P_{M, S_{\mathrm{Int}}}&= \varPhi _{\mathrm{tot}} = P_{\mathrm{tot, S}} +P_{\mathrm{tot, S_\mathrm{Int}}} \nonumber \\&=P_S - UF_{\mathrm{M,fl}} +\int _{S_{\mathrm{Int}}} da_i v_j\sigma _{\mathrm{M,ij}} + P_{M, S_{\mathrm{Int}}} \nonumber \\ \text{ or }~~~ 0&= - UF_{\mathrm{M,fl}} +\int _{S_{\mathrm{Int}}} da_i v_j\sigma _{\mathrm{M,ij}}. \end{aligned}$$

This states that the mutual power input by Marangoni stresses via the Stokes flow field is *completely* transduced via the Marangoni flow force onto the sphere, while the power input by Marangoni stresses via the Marangoni flow field itself is completely dissipated (see Eq. (A.3)).

## Legendre polynomial decomposition

In the axisymmetric decoupling approximation, we can employ a decomposition of the diffusion–advection equation into Legendre polynomials

B.1 $$\begin{aligned} {\bar{c}}(\rho ,\theta )&= \sum _{n=0}^\infty {\bar{c}}_n(\rho ) P_n(\cos \theta ), \nonumber \\ {\bar{c}}_n(\rho )&= \frac{2n+1}{2} \int _0^\pi d\theta \sin \theta P_n(\cos \theta ) {\bar{c}}(\rho ,\theta ). \end{aligned}$$

with Legendre polynomials $P_n(\cos \theta )$. Derivatives of Legendre polynomials are associated Legendre polynomials $\partial _\theta P_n(\cos \theta ) = P_n^1(\cos \theta )$. The decomposition into Legendre polynomials is advantageous as the Stokes velocity field (3a) and (3b) can be written in terms of $n=1$ polynomials only, $P_1(\cos \theta ) = \cos \theta$ and $P_1^1(\cos \theta ) = -\sin \theta$,

B.2 $$\begin{aligned} \bar{{\hat{u}}}(\rho ,\theta )&= {\bar{U}} P_1(\cos \theta ) u(\rho )= {\bar{U}} \cos \theta u(\rho ), \end{aligned}$$

B.3 $$\begin{aligned} \bar{{\hat{v}}}(\rho ,\theta )&= -{\bar{U}} P_1^1(\cos \theta ) v(\rho ) ={\bar{U}} \sin \theta v(\rho ). \end{aligned}$$

Therefore, the diffusion–advection equation (9e) only couples coefficients ${\bar{c}}_n(\rho )$ to coefficients ${\bar{c}}_{n\pm 1}(\rho )$.

Both the direct Marangoni force $F_{\mathrm{M}}$ (see (13)) and the total Marangoni force $F_{\mathrm{M,tot}}$ (see (15)) can also be written in terms of Legendre components of the concentration field at $r=a$:

B.4 $$\begin{aligned} \frac{{\bar{F}}_{\mathrm{M}}}{{\mathrm{Pe}}}&= - 2 \int _0^\pi d\theta \cos \theta {\bar{c}}(1,\theta ) \nonumber \\&= -2 \sum _{n=1}^\infty f_n {\bar{c}}_n(1) = -\pi {\bar{c}}_M(1) ~~~\text{ with } \end{aligned}$$

B.5 $$\begin{aligned} {\bar{c}}_M(\rho )&\equiv \frac{2}{\pi }\int _0^\pi d\theta \cos \theta {\bar{c}}(\rho ,\theta ) \nonumber \\&= \frac{2}{\pi }\sum _{n=1}^\infty f_n {\bar{c}}_n(\rho ) \approx {\bar{c}}_1(\rho ) + ...., \end{aligned}$$

B.6 $$\begin{aligned} {\bar{c}}_1(\rho )&= \frac{3}{2} \int _0^\pi d\theta \sin \theta \cos \theta {\bar{c}}(\rho ,\theta ), \end{aligned}$$

B.7 $$\begin{aligned} f_n&\equiv \int _0^\pi d\theta P_1(\cos \theta ) P_n(\cos \theta ) \nonumber \\&= (-1)^{(1-n)/2}\pi \frac{\varGamma \left( 1+\frac{n}{2}\right) }{\varGamma \left( \frac{n+1}{2}\right) \varGamma \left( (1-\frac{n}{2}\right) )\varGamma \left( \frac{n+3}{2}\right) } \nonumber \\&= {\left\{ \begin{array}{ll} n=2k: &{} 0 \\ n=2k+1 &{} \pi \frac{k+1/2}{k+1} \frac{(2k)!^2}{k!^416^k} = \frac{\pi }{2},~\frac{3\pi }{16},~\frac{15\pi }{128},... \end{array}\right. }, \end{aligned}$$

B.8 $$\begin{aligned} \frac{F_{\mathrm{M,tot}}}{{\mathrm{Pe}}}&= -\pi \int _1^\infty d\rho \frac{3}{4}\left( \rho ^{-1} - \rho ^{-3} \right) {\bar{c}}_M(\rho ) \nonumber \\&= -2\sum _{n=1}^\infty f_n \int _1^\infty d\rho \frac{3}{4}\left( \rho ^{-1} - \rho ^{-3} \right) {\bar{c}}_n(\rho ), \end{aligned}$$

where we used results from Ref. [45] to calculate the $f_n$ in (B.7). Advection always gives rise to an asymmetry where ${\bar{c}}(\rho ,\theta )$ is an increasing function of $\theta$; it follows that ${\bar{c}}_1(\rho )<0$ and ${\bar{c}}_M(\rho )<0$ and ${\bar{F}}_{\mathrm{M}}>0$ and ${\bar{F}}_{\mathrm{M,tot}}>0$. The coefficients are decreasing and fall off as $f_n \sim {2}/{n}$ for large *n*. This motivates to neglect all but the first $n=1$ component. For small ${\bar{U}}\ll 1$, the Legendre coefficients will scale as ${\bar{c}}_n(\rho ) \sim {\bar{U}}^n$ and this becomes an excellent approximation; for ${\bar{U}}\gg 1$, this approximation becomes worse. Therefore, Legendre decomposition is useful for ${\bar{U}}\ll 1$.

Using the decomposition (B.1) and the orthogonality relations

B.9 $$\begin{aligned} \int _0^\pi d\theta \sin \theta P_n(\cos \theta ) P_m(\cos \theta ) = \delta _{nm} \frac{2}{2n+1}, \end{aligned}$$

the diffusion–advection equation (9e) becomes

$$\begin{aligned}&\frac{2}{2n+1} \left[ \frac{1}{\rho } \partial _\rho ^2(\rho {\bar{c}}_n) - \frac{n(n+1)}{\rho ^2} {\bar{c}}_n\right] \\&= {\bar{U}} u(\rho ) \sum _m \left( \int d\theta \sin \theta P_1 P_n P_m \right) \partial _\rho {\bar{c}}_m \\&~~ - {\bar{U}} \frac{v(\rho )}{\rho } \sum _m \left( \int d\theta \sin \theta P_1^1 P_n P_m^1 \right) {\bar{c}}_m. \end{aligned}$$

The integrals on the right-hand side can be evaluated in closed form using Wigner 3-j symbols [46]:

$$\begin{aligned}&\int d\theta \sin \theta P_1 P_n P_m = 2\begin{pmatrix} 1 &{} \quad n &{} \quad m \\ 0 &{} \quad 0&{} \quad 0 \end{pmatrix}^2, \\&\int d\theta \sin \theta P_1^1 P_n P_m^1 = \\&= -2 \begin{pmatrix} 1 &{} \quad n &{} \quad m \\ 0 &{} \quad 0&{} \quad 0 \end{pmatrix} \begin{pmatrix} 1 &{} \quad n &{} \quad m \\ 1 &{} \quad 0&{} \quad -1 \end{pmatrix} \left( 2(m+1)m\right) ^{1/2}, \end{aligned}$$

which gives non-vanishing contributions only for $m=n\pm 1$. For these values, we find

$$\begin{aligned}&2\begin{pmatrix} 1 &{} \quad n &{} \quad n-1 \\ 0 &{} \quad 0&{} \quad 0 \end{pmatrix}^2 = \frac{2n}{(2n-1)(2n+1)} \\&2\begin{pmatrix} 1 &{} \quad n &{} \quad n+1 \\ 0 &{} \quad 0&{} \quad 0 \end{pmatrix}^2 = \frac{2(n+1)}{(2n+1)(2n+3)}, \\&-2 \begin{pmatrix} 1 &{} n &{} n-1 \\ 0 &{} 0&{} 0 \end{pmatrix} \begin{pmatrix} 1 &{} n &{} n-1 \\ 1 &{} 0&{} -1 \end{pmatrix} \left( 2n(n-1)\right) ^{1/2}\\&= -\frac{2n(n-1)}{(2n-1)(2n+1)}, \\&-2 \begin{pmatrix} 1 &{} \quad n &{} \quad n+1 \\ 0 &{} \quad 0&{} \quad 0 \end{pmatrix} \begin{pmatrix} 1 &{} \quad n &{} \quad n+1 \\ 1 &{} \quad 0&{} \quad -1 \end{pmatrix} \left( 2(n+1)(n+2)\right) ^{1/2}= \\&= \frac{2(n+1)(n+2)}{(2n+1)(2n+3)}. \end{aligned}$$

Finally, we obtain the diffusion–advection equation in Legendre representation Eq. (17).

## Diffusion–advection equation, perturbation theory for ${\bar{U}}\ll 1$

For small fluid velocities, ${\bar{U}}\ll 1$, we can expand about the isotropic undisturbed diffusion solution at ${\bar{U}}=0$, which is given by

C.1 $$\begin{aligned} {\bar{c}}_0^{(0)}(\rho ) = \frac{1}{\rho }~,~~{\bar{c}}_n^{(0)}(\rho ) = 0~ \text {for}~n>0 \end{aligned}$$

in Legendre representation. A first approach is a naive perturbation series Ansatz (27)

C.2 $$\begin{aligned} {\bar{c}}_n(\rho ) = \sum _{m=0}^\infty {\bar{U}}^m c_n^{(m)}(\rho ) \end{aligned}$$

for each Legendre coefficient.

It turns out that this will work only in the “inner region” $\rho < 1/{\bar{U}}$ of a solution, because in the “outer region” $\rho \gg 1/{\bar{U}}$, the convection term can no longer be treated perturbatively. In Ref. [34], a systematic expansion in inner and outer region and a matching procedure were performed for the constant concentration boundary condition (B), which we will adapt also to the constant flux boundary conditions (A) in this Appendix.

### Naive perturbation theory

We are most interested in the $n=1$ Legendre coefficient ${\bar{c}}_1(\rho )$, which will give access to both direct and total Marangoni forces at small ${\bar{U}}$ because the Legendre coefficients will scale as ${\bar{c}}_n(\rho ) \sim {\bar{U}}^n$ and the Legendre series (B.4) and (B.8) for the Marangoni forces can be terminated after $n=1$ if we are interested in the linear response for ${\bar{U}}\ll 1$. Because of the boundary conditions (A) $\partial _\rho {\bar{c}}_{n>0}(1)=0$ or (B) ${\bar{c}}_{n>0}(1)=0$, all $n>0$ modes are “generated” in Eq. (17) from lower-order terms ${\bar{c}}_{n-1}$ on the right-hand sides:

C.3 $$\begin{aligned}&\frac{1}{\rho } \partial _\rho ^2(\rho {\bar{c}}_0) = {\bar{U}} \left( u(\rho ) \frac{1}{3} \partial _\rho {\bar{c}}_{1}- \frac{v(\rho )}{\rho } \frac{2}{3} {\bar{c}}_{1} \right) , \nonumber \\&\left[ \frac{1}{\rho } \partial _\rho ^2(\rho {\bar{c}}_1) - \frac{2}{\rho ^2} {\bar{c}}_1 \right] = {\bar{U}} u(\rho ) \left( \partial _\rho {\bar{c}}_{0} + \frac{2}{5} \partial _\rho {\bar{c}}_{2} \right) \nonumber \\&~~~~~~~~~~~~+\, {\bar{U}} \frac{v(\rho )}{\rho } \left( - \frac{6}{5} {\bar{c}}_{2} \right) , \nonumber \\&... \nonumber \\&{\bar{c}}_0(\infty ) = {\bar{c}}_\infty ~,~~ {\bar{c}}_{n>0}(\infty )=0, \nonumber \\&\text{(A) } \text{ constant } \text{ flux: }~~ \partial _\rho {\bar{c}}_0(1) = -1~,~~\partial _\rho {\bar{c}}_{n>0}(1) = 0, \nonumber \\&\text{(B) } \text{ constant } \text{ concentration: }~~ {\bar{c}}_0(1) = 1~,~~{\bar{c}}_{n>0}(1) = 0. \end{aligned}$$

This hierarchy results in

C.4 $$\begin{aligned} {\bar{c}}_n(\rho ) = \sum _{m=n}^\infty {\bar{U}}^m {\bar{c}}_n^{(m)}(\rho ), \end{aligned}$$

i.e., the leading orders are ${\bar{c}}_n(\rho ) \propto {\bar{U}}^n$.

In a naive perturbative approach, we start with

$$\begin{aligned} {\bar{c}}_0^{(0)}(\rho ) = \frac{1}{\rho }~,~~ {\bar{c}}_n^{(0)} =0 ~~n\ge 1. \end{aligned}$$

for both boundary conditions. We obtain in first order

$$\begin{aligned}&\left[ \frac{1}{\rho } \partial _\rho ^2(\rho {\bar{c}}_1^{(1)}) - \frac{2}{\rho ^2} {\bar{c}}_1^{(1)} \right] = u(\rho ) \left( \partial _\rho {\bar{c}}_{0}^{(0)} \right) . \end{aligned}$$

Solving for ${\bar{c}}_1^{(1)}$ and regularizing using a finite system $\rho <R$ (i.e., the boundary conditions ${\bar{c}}_0({\bar{R}}) = 0$ and ${\bar{c}}_{n>0}({\bar{R}})=0$), we find up to linear order in ${\bar{U}}$

$$\begin{aligned}&\text{(A) } \text{ constant } \text{ flux: }~~\\&{\bar{c}}_1(\rho ) = -\frac{1}{2}{\bar{\beta }} \frac{1}{\rho ^2}+ {\bar{U}}\left( \frac{1}{8}\frac{1}{\rho ^3} - \frac{9}{16} \frac{1}{\rho ^2} +\frac{3}{4} \frac{1}{\rho } - \frac{1}{2} \right) + O\!\left( \frac{1}{{\bar{R}}}\right) , \\&\text{(B) } \text{ constant } \text{ concentration: }~~\\&{\bar{c}}_1(\rho ) = {\bar{c}}_{S,1} \frac{1}{\rho ^2}+ {\bar{U}} \left( - \frac{3}{8}\frac{1}{\rho ^2} +\frac{1}{8}\frac{1}{\rho ^3} + \frac{3}{4} \frac{1}{\rho } - \frac{1}{2} \right) + O\!\left( \frac{1}{{\bar{R}}}\right) , \end{aligned}$$

which fulfills the more general explicitly symmetry-breaking flux boundary condition $\partial _\rho {\bar{c}}_1(1)= {\bar{\beta }}$ (see Eq. (18)) with ${\bar{\beta }}=0$ as constant flux (A) or concentration boundary condition ${\bar{c}}_1(1) = {\bar{c}}_{S,1}$ see Eq. (19)) with ${\bar{c}}_{S,1}=0$ as constant concentration (B). These more general boundary conditions just add a zeroth-order $U^0$-term to the $n=1$ component ${\bar{c}}_1(\rho )$.

We obtain for flux boundary conditions (A)

C.5 $$\begin{aligned} \frac{{\bar{F}}_{\mathrm{M}}}{\pi \mathrm{Pe}}&\approx -{\bar{c}}_1(1) =\frac{1}{2}{\bar{\beta }}+ \frac{3}{16} {\bar{U}}, \ \end{aligned}$$

C.6 $$\begin{aligned} \frac{{\bar{F}}_{\mathrm{M,tot}}}{\pi \mathrm{Pe}}&= -\int _1^\infty d\rho \frac{3}{4}\left( \rho ^{-1} - \rho ^{-3} \right) c_1(\rho ) \nonumber \\&= \frac{3}{32}{\bar{\beta }}- \frac{1081}{1280} {\bar{U}} +\frac{3}{8} {\bar{U}}\ln {\bar{R}}. \end{aligned}$$

This means that the direct Marangoni force ${\bar{F}}_{\mathrm{M}}$ is linear with a finite linear coefficient for large ${\bar{R}}$, whereas the total Marangoni force ${\bar{F}}_{\mathrm{M,tot}}$ has a logarithmically diverging linear coefficient for large ${\bar{R}}$ (stemming from the $\rho$-independent contribution $c_1^{(1)}(\rho )= -1/2+...$). The contribution from explicit symmetry breaking (${\bar{\beta }}$) to the direct Marangoni force is always *weakened* by the presence of Marangoni flows.

For concentration boundary conditions (B), we have

C.7 $$\begin{aligned} \frac{{\bar{F}}_{\mathrm{M}}}{\pi \mathrm{Pe}}&\approx -{\bar{c}}_1(1)=-{\bar{c}}_{S,1} + O({\bar{U}}^3), \end{aligned}$$

C.8 $$\begin{aligned} \frac{{\bar{F}}_{\mathrm{M,tot}}}{\pi \mathrm{Pe}}&= -\int _1^\infty d\rho \frac{3}{4}\left( \rho ^{-1} - \rho ^{-3} \right) c_1(\rho ) \nonumber \\&= -\frac{3}{8}{\bar{c}}_{S,1} - \frac{563}{320} {\bar{U}} +\frac{3}{4} {\bar{U}}\ln {\bar{R}}. \end{aligned}$$

This means that the direct Marangoni force ${\bar{F}}_{\mathrm{M}}$ (see(13)) is only present for explicit symmetry breaking (${\bar{c}}_{S,1}\ne 0$), whereas the total Marangoni force ${\bar{F}}_{\mathrm{M,tot}}$ [see (15)] has a logarithmically diverging linear coefficient for large ${\bar{R}}$, which is identical to the constant flux case. The contribution from explicit symmetry breaking (${\bar{c}}_{S,1}$) to the direct Marangoni force is always *weakened* by the presence of Marangoni flows.

### Matching procedure

In order to go beyond naive perturbation theory for the constant flux boundary condition (A) in the fluid velocity window $1/R <{\bar{U}}\ll 1$, we must adapt the matching procedure that was developed for constant-*c* boundary conditions (B) in Ref. [34]. This matching procedure employs both the real space representation and a Legendre decomposition. We start from the diffusion–advection equation (9e) (neglecting $\bar{\varvec{v}}_{\mathrm{M}}$ in the decoupled limit) in the original angular representation but in dimensionless form for ${\bar{c}} = {\bar{c}}(\rho ,\theta )$ or ${\bar{c}} = {\bar{c}}(\rho ,\eta )$ ($\eta \equiv \cos \theta$).^2^

A perturbation expansion

C.9 $$\begin{aligned} {\bar{c}}(\rho ,\eta )&= \sum _{n=0}^\infty f_n({\bar{U}}) {\bar{c}}^{(n)}(\rho ,\eta ), \nonumber \\&\lim _{{\bar{U}}\rightarrow 0} \frac{f_{n+1}}{f_n} =0 ~~\text{ and }~~ f_0({\bar{U}})=1 \end{aligned}$$

with constant flux boundary conditions

C.10 $$\begin{aligned} \partial _\rho {\bar{c}}^{(0)}(1,\eta ) = -1+{\bar{\beta }}\eta ~, ~~\partial _\rho {\bar{c}}^{(n\ge 1)}(0,\eta ) = 0\nonumber \\ \end{aligned}$$

is used in the “inner region” $\rho < 1/{\bar{U}}$. In the “outer region” $\rho \gg 1/{\bar{U}}$, the convection term can no longer be treated perturbatively, regardless how small ${\bar{U}}$ is [34]. Here, we rescale $\sigma \equiv \rho {\bar{U}}$ and ${\bar{C}}(\sigma ,\eta ) = {\bar{c}}(\sigma /{\bar{U}},\eta )$ to obtain an equation

C.11 $$\begin{aligned} \varvec{\nabla }_\sigma ^2 {\bar{C}}&= \eta u\left( \frac{\sigma }{{\bar{U}}}\right) \partial _\sigma {\bar{C}} - (1-\eta ^2) \frac{v\left( \frac{\sigma }{{\bar{U}}}\right) }{\sigma } \partial _\eta {\bar{C}}\nonumber \\ \end{aligned}$$

and use an expansion

C.12 $$\begin{aligned} {\bar{C}}(\sigma ,\eta ) = \sum _{n=0}^\infty F_n({\bar{U}}) {\bar{C}}^{(n)}(\sigma ,\eta ) ~,~~ \lim _{{\bar{U}}\rightarrow 0} \frac{F_{n+1}}{F_n} =0\nonumber \\ \end{aligned}$$

with outer boundary conditions

C.13 $$\begin{aligned} {\bar{C}}^{(n)}(\infty ,\eta ) = 0. \end{aligned}$$

Both expansions have to match at a large but finite $\rho$, such that

C.14 $$\begin{aligned} {\bar{c}}(\rho \rightarrow \infty ,\eta ) = {\bar{c}}(\sigma /{\bar{U}}\rightarrow \infty ,\eta )= {\bar{C}}(\sigma \rightarrow 0,\eta ) \nonumber \\ \end{aligned}$$

in all orders in ${\bar{U}}$ and as a function of $\eta$ or in all Legendre coefficients.

Plugging the expansions into the inner and outer equations (9e) and (C.11), respectively, and isolating the ${\bar{U}}^0$-terms, the functions ${\bar{c}}^{(0)}(\rho ,\eta )$ and ${\bar{C}}^{(0)}(\sigma ,\eta )$ have to fulfill

$$\begin{aligned} \bar{\varvec{\nabla }}^2 {\bar{c}}^{(0)}&=0, \\ \varvec{\nabla }_\sigma ^2 {\bar{C}}^{(0)}&= -\eta \partial _\sigma {\bar{C}}^{(0)} - (1-\eta ^2) \frac{1}{\sigma } \partial _\eta {\bar{C}}^{(0)} \end{aligned}$$

and constant flux boundary conditions, resulting in

$$\begin{aligned} {\bar{c}}^{(0)}(\rho ,\eta )&= B_0 + \frac{1}{\rho } + {\bar{\beta }}\rho P_1(\eta ) \\&~~ + \sum _{k=1}^\infty B_k (\rho ^{-k-1} + \frac{k+1}{k}\rho ^k ) P_k(\eta ) \end{aligned}$$

and (unchanged from the constant concentration case [34])

$$\begin{aligned} {\bar{C}}^{(0)}(\sigma ,\eta )&= e^{-\sigma \eta /2} \left( \frac{\pi }{\sigma }\right) ^{1/2} \sum _{k=0}^\infty C_k K_{k+1/2}(\sigma /2)P_k(\eta ) \end{aligned}$$

with the modified Bessel functions

$$\begin{aligned} K_{k+1/2}\left( \frac{\sigma }{2}\right)&= \left( \frac{\pi }{\sigma }\right) ^{1/2}e^{-\sigma /2} \sum _{m=0}^k\frac{(k+m)!}{(k-m)!m! \sigma ^m}. \end{aligned}$$

Matching the zeroth-order contributions according to (C.14) means

$$\begin{aligned} {\bar{c}}^{(0)}(\sigma /{\bar{U}},\eta )&= B_0 +\frac{{\bar{U}}}{\sigma } + {\bar{\beta }}\frac{\sigma }{{\bar{U}}} P_1(\eta )\\&~~ + \sum _{k=1}^\infty B_k \frac{k+1}{k}\frac{\sigma ^k}{{\bar{U}}^k} P_k(\eta ) \end{aligned}$$

has to match

$$\begin{aligned} F_0({\bar{U}}) {\bar{C}}^{(0)}(\sigma ,\eta )&= F_0({\bar{U}})\frac{\pi }{\sigma } \left[ 1+ \frac{\sigma }{2}(-\eta -1) + ...\right] \\&~~\times \sum _{k=0}^\infty C_k \frac{(2k)!}{k! \sigma ^k}P_k(\eta ) \end{aligned}$$

for small $\sigma$ and ${\bar{U}} \rightarrow 0$ for all Legendre coefficients *k*. This yields

$$\begin{aligned} F_0({\bar{U}})&= {\bar{U}}~,~~C_0 = \frac{1}{\pi }~,~~ B_0 = -{\bar{U}}/2 \rightarrow 0~,~~B_1 = -{\bar{\beta }}/2 \\ B_{k\ge 2}&=0~,~~ C_{k\ge 1}=0. \end{aligned}$$

$B_0 = -{\bar{U}}/2$ gives an $O({\bar{U}})$-contribution to ${\bar{c}}^{(0)}$, which should be attributed to the next order ${\bar{c}}^{(1)}$. The final result for the zeroth-order contributions is

$$\begin{aligned} {\bar{c}}^{(0)}(\rho ,\eta )&= \frac{1}{\rho } -\frac{{\bar{\beta }}}{2\rho ^2}\eta , \\ {\bar{C}}^{(0)}(\sigma ,\eta )&= \frac{1}{\sigma } \exp \left( \frac{\rho }{2}(-\eta -1) \right) , \end{aligned}$$

which is unchanged from the constant *c* case [34] (apart from the different $\theta$-convention leading to $\eta \rightarrow -\eta$).

Assuming $f_1({\bar{U}}) = {\bar{U}}$ and $F_1({\bar{U}}) = {\bar{U}}^2$, plugging the expansions including the already calculated zeroth-order contributions into the inner and outer equations (9e) and (C.11), respectively, and isolating the ${\bar{U}}^1$-terms, we find that the first-order contributions ${\bar{c}}^{(1)}(\rho ,\eta )$ and ${\bar{C}}^{(1)}(\sigma ,\eta )$ have to fulfill the same equations as for the constant *c* case [34],

$$\begin{aligned} \bar{\varvec{\nabla }}^2 {\bar{c}}^{(1)}&= u(\rho ) P_1(\eta ) \partial _\rho {\bar{c}}^{(0)} =\left[ 1 - \frac{3}{2} \frac{1}{\rho } + \frac{1}{2} \frac{1}{\rho ^3} \right] \frac{\eta }{\rho ^2}, \\ \varvec{\nabla }_\sigma ^2 {\bar{C}}^{(1)}&= -\eta \partial _\sigma {\bar{C}}^{(1)} - (1-\eta ^2) \frac{1}{\sigma } \partial _\eta {\bar{C}}^{(1)}\\&~~ +\frac{3}{2}\frac{\eta }{\sigma }\partial _\sigma {\bar{C}}^{(0)} + \frac{3}{4}\frac{1-\eta ^2}{\sigma } \partial _\eta {\bar{C}}^{(0)}. \end{aligned}$$

A particular solution for ${\bar{c}}^{(1)}$ is

$$\begin{aligned} {\bar{c}}_p^{(1)} = -\left( \frac{1}{2} - \frac{3}{4\rho } - \frac{1}{8\rho ^3}\right) \eta ; \end{aligned}$$

the full solution that fulfills the flux boundary condition $\partial _\rho {\bar{c}}^{(1)}(0,\eta ) = 0$ is

$$\begin{aligned} {\bar{c}}^{(1)}&= B_0 + \left[ (2B_1 - \frac{9}{8} ) \rho + \frac{B_1}{\rho ^2} - \left( \frac{1}{2} - \frac{3}{4\rho } - \frac{1}{8\rho ^3}\right) \right] \eta \\&~~ + \sum _{k=2}^\infty B_k (r^{-k-1} + \frac{k+1}{k}r^k ) P_k(\eta ). \end{aligned}$$

Now, we can determine all $B_k$ by matching all contributions up to $O({\bar{U}})$ according to (C.14),

$$\begin{aligned}&{\bar{c}}^{(0)} (\sigma /{\bar{U}}\rightarrow \infty ,\eta )+ {\bar{U}} {\bar{c}}^{(1)} (\sigma /{\bar{U}}\rightarrow \infty ,\eta ) \\&~~ = {\bar{U}} {\bar{C}}^{(0)}(\sigma \rightarrow 0,\eta ) \end{aligned}$$

resulting in

$$\begin{aligned} B_0 = -1/2~,~~ B_1 = -9/16~,~~ B_{k\ge 1}=0. \end{aligned}$$

$B_0=-1/2$ for ${\bar{c}}^{(1)}$ is indeed equivalent to our above $B_0 = -{\bar{U}}/2$ for ${\bar{c}}^{(0)}$; now, this term is consistently attributed to ${\bar{c}}^{(1)}$. All in all, we have up to $O({\bar{U}})$

C.15 $$\begin{aligned} {\bar{c}}^{(0)}+ {\bar{U}} {\bar{c}}^{(1)}&= \frac{1}{\rho }-\frac{{\bar{\beta }}}{2\rho ^2}\eta \nonumber \\&~~ +{\bar{U}}\left[ -\frac{1}{2} - \left( \frac{1}{2} - \frac{3}{4\rho } + \frac{9}{16\rho ^2}-\frac{1}{8\rho ^3} \right) \eta \right] . \end{aligned}$$

We can use this result to calculate the first Legendre coefficient at the boundary $-{\bar{c}}_1(\rho =1)$, which gives

C.16 $$\begin{aligned} -{\bar{c}}_1(1) = -\frac{3}{2} \int _{-1}^1 d\eta \eta \left. ({\bar{c}}^{(0)}+ {\bar{U}} {\bar{c}}^{(1)})\right| _{\rho =1} = \frac{{\bar{\beta }}}{2} + \frac{3}{16} {\bar{U}}\nonumber \\ \end{aligned}$$

in complete agreement with our above result (C.5) from naive perturbation theory.

We continue with the next order $C^{(1)}(\sigma ,\eta )$ of the outer solution. Here, we obtain the same result as for the constant concentration case [34],

$$\begin{aligned} {\bar{C}}^{(1)}&= e^{-\sigma \eta /2} \left[ \left( \frac{\pi }{\sigma }\right) ^{1/2} \sum _{k=0}^\infty C_k^* K_{k+1/2}(\sigma /2)P_k(\eta )\right. \\&~~~~~~~~\left. + \sum _{i=0}^2 {\tilde{R}}_i(\sigma ) P_i(\eta )\right] \end{aligned}$$

with functions ${\tilde{R}}_i(\sigma ) = (-1)^iR_i(\sigma )$ with the functions $R_i(\sigma )$ from [34] (because of the different $\theta$-convention leading to $\eta \rightarrow -\eta$).

In the variable $\sigma \equiv \rho {\bar{U}}$, in which the matching to the outer solution is performed, the inner solution (C.15) becomes

C.17 $$\begin{aligned} {\bar{c}}^{(0)}+ {\bar{U}} {\bar{c}}^{(1)}&= {\bar{U}} \left[ \frac{1}{\sigma } +\frac{1}{2}(-\eta -1) \right] +{\bar{U}}^2 \frac{1}{\sigma } \frac{3}{4} \eta + ... \end{aligned}$$

All terms $\sigma ^{-m}$ ($m\ge 1$) of the inner solution from ${\bar{c}}^{(n\ge 2)}$ are of higher order and, thus, at least $O({\bar{U}}^3)$; only an additional constant term $O({\bar{U}}^2)$ from ${\bar{c}}^{(2)}$ is possible. Therefore, in order to match (C.17), all terms $\sigma ^{-m}$ for $m\ge 2$ of the outer solution ${\bar{C}}^{(1)}$ have to be zero and the $\sigma ^{-1}$-term has to equal $\sigma ^{-1}\frac{3}{4}\eta$. We conclude that

$$\begin{aligned} C_{k\ge 3}^*&=0,\\ C_2^*&= -\frac{1}{4\pi }(3-\ln \gamma )~,~~ C_1^* = -\frac{3}{4\pi }(1-\ln \gamma ),\\ C_0^*&= \frac{1}{2\pi } \ln \gamma \end{aligned}$$

(where $\ln \gamma =0.577216$ is the Euler constant), i.e., only $C_0^*$ is different from the constant concentration results of [34] ($C_1^*$ has an additional minus sign because of $\eta \rightarrow -\eta$). The resulting outer solution

C.18 $$\begin{aligned}&{\bar{U}} {\bar{C}}^{(0)} + {\bar{U}}^2 {\bar{C}}^{(1)} =\nonumber \\&= \frac{{\bar{U}}}{\sigma }\exp \left( \frac{\sigma }{2}(-\eta -1) \right) +{\bar{U}}^2\left( -\frac{\ln \sigma }{2} + \left( \frac{1}{2}-\frac{\ln \gamma }{4} \right) \right) \nonumber \\&~~ - {\bar{U}}^2\left( -\frac{3}{4\sigma }\ln \gamma -\frac{3}{16}(\ln \gamma -1)\right) \eta \nonumber \\&\approx \frac{{\bar{U}}}{\sigma }\left[ 1-\frac{\sigma }{2}+\frac{\sigma ^2}{6} - \left( \frac{\sigma }{2}-\frac{\sigma ^2}{4}\right) \eta \right] \nonumber \\&~~ +{\bar{U}}^2\left( -\frac{\ln \sigma }{2} + \left( \frac{1}{2}-\frac{\ln \gamma }{4} \right) \right) P_0(\eta ) \nonumber \\&~~ + {\bar{U}}^2\left( \frac{3}{4\sigma }\ln \gamma -\frac{3}{16}(\ln \gamma -1)\right) P_1(\eta ) \end{aligned}$$

contains terms ${\bar{U}}^2 \sigma ^0P_0(\eta )$ and ${\bar{U}}^2\ln \sigma P_0(\eta )$, which suggests that the second-order contribution ${\bar{c}}^{(2)}$ to the inner solution should also contain constant terms ${\bar{U}}^2$ and ${\bar{U}}^2\ln {\bar{U}}$ in order to match the outer solution.

Now, we turn to this contribution ${\bar{c}}^{(2)}(\rho ,\eta )$. Plugging the expansion (C.9) including the already calculated (C.15) up to the first order into the inner equation (9e) and isolating the ${\bar{U}}^2$-terms, we find

$$\begin{aligned} \bar{\varvec{\nabla }}^2 {\bar{c}}^{(2)}&= u(\rho ) P_1(\eta ) \partial _\rho {\bar{c}}^{(1)} - \frac{v(\rho )}{\rho } (1-\eta ^2) \partial _\eta {\bar{c}}^{(1)}. \end{aligned}$$

Inserting the first-order part from (C.15) on the right-hand side, we finally obtain

$$\begin{aligned} \bar{\varvec{\nabla }}^2 {\bar{c}}^{(2)}&= Z_0(\rho ) P_0(\eta ) + Z_2(\rho ) P_2(\eta ) ~~~\text{ with } \\ Z_0(\rho )&= \frac{1}{3\rho } -\frac{1}{2\rho ^2} +\frac{23}{96\rho ^4} +\frac{1}{16\rho ^5}-\frac{9}{32\rho ^6}+\frac{1}{12\rho ^7},\\ Z_2(\rho )&= -\frac{1}{3\rho } +\frac{7}{4\rho ^2}-\frac{9}{4\rho ^3} +\frac{175}{96\rho ^4}\\&~~ -\frac{5}{16\rho ^5}-\frac{9}{32\rho ^6}+\frac{5}{48\rho ^7}. \end{aligned}$$

A $P_1(\eta )$-term is absent on the right hand side because ${\bar{c}}^{(1)}$ from (C.15) contains no $P_0(\eta )$-component but is a pure $P_1(\eta )$-term. In order to obtain the first two Legendre components $k=0,1$ of ${\bar{c}}^{(2)}$, we thus have to solve

$$\begin{aligned} \partial _\rho ^2 {\bar{c}}^{(2)} + \frac{2}{\rho }{\bar{c}}^{(2)}&= Z_0(\rho ) \end{aligned}$$

and find

$$\begin{aligned} {\bar{c}}^{(2)}&= B_0 + L_0(\rho ) +\eta B_1\left( \rho + \frac{1}{2\rho ^2}\right) + P_2(\eta ) \left( ...\right) + ..., \\ L_0(\rho )&= \frac{1}{240\rho ^5}-\frac{3}{128\rho ^4} +\frac{1}{48\rho ^3}+\frac{23}{192}{\rho ^2}\\&~~ -\frac{9}{16\rho } +\frac{527}{1920}+\frac{\rho }{6}-\frac{1}{2}\ln \rho . \end{aligned}$$

Using $\rho = \sigma /{\bar{U}}$ and matching with ${\bar{U}} {\bar{C}}^{(0)} + {\bar{U}}^2 {\bar{C}}^{(1)}$ from (C.18) gives

$$\begin{aligned} B_0&= \left( \frac{1}{2}-\frac{\ln \gamma }{4} \right) -\frac{527}{1920} ~,~~ B_1 = 1/4. \end{aligned}$$

Up to the second order, we obtain from (C.15) and ${\bar{c}}^{(2)}$ a $P_1(\eta )$-contribution

$$\begin{aligned} {\bar{c}}_1(\rho )&= -\frac{{\bar{\beta }}}{2\rho ^2} -{\bar{U}} \left( \frac{1}{2} - \frac{3}{4\rho } + \frac{9}{16\rho ^2}-\frac{1}{8\rho ^3} \right) \\&~~ +{\bar{U}}^2 \frac{1}{4}\left( \rho + \frac{1}{2\rho ^2}\right) + O({\bar{U}}^3). \end{aligned}$$

We can directly obtain the value ${\bar{c}}_1(1)$ of the first Legendre coefficient at the surface as

C.19 $$\begin{aligned} -{\bar{c}}_1(\rho =1)&= \frac{{\bar{\beta }}}{2} + \frac{3}{16}{\bar{U}} - \frac{3}{4}{\bar{U}}^2 + O({\bar{U}}^3). \end{aligned}$$

The leading $O({\bar{U}})$-term agrees with the naive perturbation expansion results (C.5). The matching procedure gives, however, a non-vanishing second-order contribution $O({\bar{U}}^2)$, which is absent in the naive perturbation expansion. The matching to the outer solution, which features a $P_1(\eta )$-contribution in second order, see (C.18), enforces this term. Matching is required for ${\bar{R}}\gg 1/{\bar{U}}$. For ${\bar{U}}\ll 1/{\bar{R}}$, the completely symmetric outer boundary condition ${\bar{c}}(\rho ={\bar{R}})=0$ suppresses this term and the naive perturbation expansion for ${\bar{c}}_1(\rho )$ only contains odd powers of ${\bar{U}}$.

For constant concentration boundary conditions (B), we can directly employ the results from Ref. [34] to obtain up to the second order a $P_1(\eta )$-contribution

$$\begin{aligned} {\bar{c}}_1(\rho )&= \frac{{\bar{c}}_{S,1}}{\rho ^2} -{\bar{U}} \left( \frac{1}{2} - \frac{3}{4\rho } + \frac{3}{8\rho ^2}-\frac{1}{8\rho ^3} \right) \\&~~ -{\bar{U}}^2 \left( \frac{7}{16\rho ^2} - \frac{1}{4}\rho + \frac{1}{4} - \frac{3}{8\rho } - \frac{1}{16\rho ^3} \right) + O({\bar{U}}^3) \end{aligned}$$

leading to

$$\begin{aligned} -{\bar{c}}_1(1)&= -{\bar{c}}_{S,1} + O({\bar{U}}^3). \end{aligned}$$

This agrees with the naive perturbation expansion result (C.7).

There is, however, an important difference in evaluating the $\rho$-integrals in Eq. (C.6) and (C.8) in order to calculate the total Marangoni force as compared to the naive perturbation theory. Also, these integrals have to be divided into inner and outer region in the framework of the matching procedure, which essentially provides an upper cutoff ${\bar{R}}\sim 1/{\bar{U}}$ to the otherwise unchanged inner region. Therefore, contribution $\propto {\bar{U}}\ln {\bar{R}}$ are to be replaced by corresponding contributions $\propto -{\bar{U}}\ln {\bar{U}}$.

## Footnotes

1 In many publications on the diffusion–advection problem, such as Refs. [33–35] but also in Refs. [20, 28, 29, 31, 40], ${\bar{U}}$ is called the Peclet number. Here, we define the Peclet number as $\mathrm{Pe} \equiv {\bar{U}}_{\alpha }$, i.e., by the characteristic velocity ${\bar{U}}_{\alpha } = {\kappa \alpha a}/{D \mu }$ for constant flux boundary conditions. We define it as $\mathrm{Pe} \equiv {\bar{U}}_{c_s} = {\kappa c_S a}/{D \mu }$ for constant concentration boundary conditions. ${\bar{U}}_{\alpha }$ and ${\bar{U}}_{c_s}$ are characteristic velocities, where a typical direct Marangoni force $F_{\mathrm{M}} \sim \kappa a^2 \partial _rc(r=a)\sim \kappa a^2\alpha /D$ for constant flux boundary conditions or $F_{\mathrm{M}} \sim \kappa a c(r=a)\sim \kappa c_S a/D$ for constant concentration boundary conditions is balanced by the typical Stokes drag force $F_D \sim \mu a U$. The Peclet number is a dimensionless measure of propulsion strength with these definitions.
